# Midnight/midday-synchronized expression of cryptochrome genes in the eyes of three teleost species, zebrafish, goldfish, and medaka

**DOI:** 10.1186/s40851-022-00192-4

**Published:** 2022-06-07

**Authors:** Marika Nakagawa, Keiko Okano, Yuya Saratani, Yosuke Shoji, Toshiyuki Okano

**Affiliations:** Department of Electrical Engineering and Bioscience, Graduate School of Sciences and Engineering, Waseda University, TWIns, Wakamatsucho 2-2, Shinjuku-Ku, Tokyo, 162-8480 Japan

**Keywords:** Cryptochrome, Circadian clock, Photoperiodicity, Sun compass, Quantitative RT-PCR, Eye, Zebrafish, Goldfish, Medaka

## Abstract

Photoperiodic responses are observed in many organisms living in the temperate zones. The circadian clock is involved in photoperiodic time measurement; however, the underlying molecular mechanism for detection of the day length remains unknown. We previously compared the expression profiles of the *Cryptochrome(Cry)* genes in the zebrafish eye and reported that *Cry1ab* has a double peak with variable expression duration depending on the photoperiod. In this study, to understand commonalities and differences in the photoperiodic responses of ocular *Cry* genes, we identified *Cryptochrome* genes in two other teleost species, goldfish and medaka, living in temperate zones, and measured ocular *Cry* mRNA levels in all of the three species, under different photoperiods (long-day [14 h light: 10 h dark] and short-day [10 h light: 14 h dark] and in constant darkness. *Cry1ab* mRNA levels did not show dual peaks in goldfish or medaka under the examined conditions; however, the mRNA expression profiles of many *Cry*s were altered in all three species, depending on the day length and light condition. Based on their expression profiles, *Cry* mRNA peaks were classified into three groups that better synchronize to sunrise (light-on), midnight/midday (middle points of the dark/light periods), or sunset (light-off). These results suggest the presence of multiple oscillators that oscillate independently or a complex oscillator in which *Cry* expression cycles change in a photoperiod-dependent manner in the eye.

**Supplementary Information:**

The online version contains supplementary material available at 10.1186/s40851-022-00192-4.

## Background

Photoperiodism is the response of living organisms to seasonal changes in day length. Organisms living in temperate zones use day length for detecting seasonal changes, because the day-length changes stably depending on the season, while the temperature is greatly affected by the weather. Photoperiodic responses in animals include gonadal developments [1], migration [2], replacement of feathers [3], and hibernation [4]. A critical step in the photoperiodic response is photoperiodic time measurement (PTM) that detects the day length [5, 6]. The PTM is known to be closely associated with the circadian clock; however, the underlying molecular mechanism remains to be understood.

The circadian clock comprises a feedback loop mechanism, regulated by the expression of the clock genes [7]. In vertebrate circadian clocks, PERIOD (PER) and CRYPTOCHROME (CRY) inhibit transcription from the E-box elements of *Per* and *Cry* genes, by suppressing the transactivation activity of BMAL-CLOCK [8, 9]. Mammals have two CRY paralogs, CRY1 and CRY2, both of which are transcriptional repressors of the circadian clock. In non-mammalian vertebrates, CRY1 and CRY2 not only function as clock factors, but also as blue light photoreceptors [10], similarly to CRY in plants and invertebrates [11, 12]. In addition, non-mammals possess CRY4 as the third paralog, which is presumed to be a photoreceptive magnetoreceptor or a blue light sensor [13–16]. In addition to *Cry2* and *Cry4*, zebrafish have four *Cry1* paralogs (*Cry1aa/1ab/1ba/1bb* [17], termed as *zCry1a/1b/2a/2b* in a previous report [18]) probably resulting from whole-genome duplications [19, 20]. Five of the six *zCry* paralogs, except for *zCry4* (*zCry1aa/1ab/1ba/1bb/2*), negatively regulate E-box-mediated transactivation via BMAL-CLOCK [21], presuming their circadian-clock-related functions. However, their functional differentiation remains unknown.

To understand the functional differentiation of the teleost *Cry* paralogs, we analyzed the expression of the *Cry* genes in zebrafish [22]. Among the six *zCry*s examined, *zCry1ab* showed dual peaks, one in the morning and the other at the end of the light period, in an eye-specific manner. Each of these peaks is synchronized at midnight and at the end of light as the day length changes, and therefore the *zCry1ab* expression profile fluctuates in response to the day length. These results led us to propose a mechanism for the discrimination of day length in which the states of multiple circadian oscillators vary depending on the day length, resulting in the photoperiodic expression of *zCry1ab* [22]. Such a mechanism may control the day-length-dependent changes in the expression of the melanopsin gene in the zebrafish eye [23]. To further strengthen the support for this hypothesis and establish its universality, it is important to know [i] whether *zCry* genes other than *zCry1ab* have day-length dependency in the oscillation state, i.e. peak shifts and separations and [ii] whether the photoperiod-dependent fluctuations in gene expression seen in *zCrys* are also found in the eyes of other photoperiodic fish species living in the temperate zones.

In this study, we focused on three teleost species, zebrafish, goldfish, and Japanese medaka (*Oryzias latipes* and *Oryzias sakaizumii*, simply termed “medaka” in the present study). Goldfish is a freshwater fish that originated from crucian carp, has been bred in Asia for more than 2000 years, and is now found worldwide, with a few exceptions [24, 25]. Fish species belonging to the *Oryzias* genus are native to Japan, South Korea, and China; they inhabit paddy fields, ponds, canals, and rivers [26]. In both goldfish and medaka, genome sequences are well-characterized and clear seasonal variations are observed: sexual maturation of female goldfish is observed in 16L8D (16 h light: 8 h dark) but it was suppressed in 12L12D (12 h light: 12 h dark) [27], and the reproductive activity of medaka begins in spring and ends in autumn [28]. Although photoperiodic responses had been less characterised in zebrafish, the breeding season of zebrafish is reportedly between April and August [29]. Considering these facts, this study analyzed the expression of *Cry* in the eye of the three fish species, under the long-day (LD; 14 h light: 10 h dark), short-day (SD; 10 h light: 14 h dark), and constant dark (DD) after the entrainment under LD or SD conditions (LD-DD, SD-DD). Based on those expression profiles, we discuss the existence and physiological significance of *Cry* gene expression oscillations synchronized with the midpoints of the light and dark periods (noon and midnight). Because it was previously unknown whether photoperiodic responses known to occur in the gene expression of *zCry*s are found in other fish species, the objectives of this study could help elucidate the previously unknown mechanisms of PTM and day-length discrimination.

## Results

### Identification and phylogenetic analysis of goldfish and medaka cryptochrome genes

**Fig. 1:**
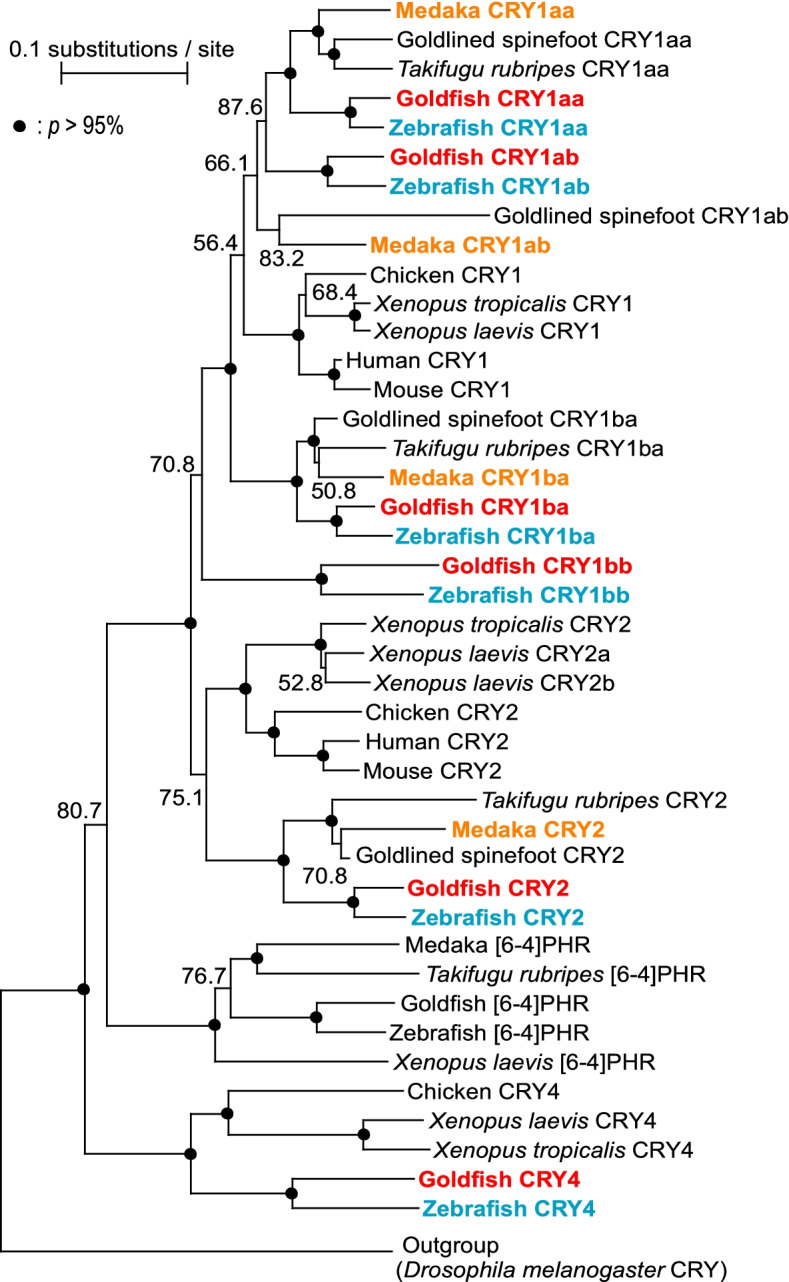
Molecular phylogenetic tree of the CRY / Photolyase family. A molecular phylogenetic tree was constructed by ClustalW (ver. 2.1). The accession numbers for all genes are shown in Table S1. Bootstrap probabilities (*p*) of 95% or more are indicated using closed circles, and numbers below 95% are shown. In this study, we investigated the expression of *Cry* mRNA in zebrafish (blue), goldfish (red), and medaka (orange)

We found six different goldfish *Cryptochrome* (*gCry*) gene cDNA sequences in the GenBank database (Table S1). Molecular phylogenetic analysis of the six types of *Cry* in zebrafish (*zCry1aa/1ab/1ba/1bb/2/4*) and four types of *Cry* in medaka [30] (*OlCry1aa/1ab/1ba/2*) resulted in identification of six types of *Cry* (*gCry1aa/1ab/1ba/1bb/2/4*) in goldfish (Fig. 1, Table S1). We found no *Cry1bb* or *Cry4* orthologs in the medaka genome. Focusing on *Cry* orthologs (*Cry1aa/1ab/1ba/2*) common to all the three species, we found that, except for *Cry1ab*, which did not show high confidence in the molecular phylogenetic tree (87.6%), goldfish and zebrafish clustered together, resulting in formation of an independent monophyletic clade, consistent with the species divergence.

### Daily and circadian variations in the expression of zebrafish *Cry*

**Fig. 2:**
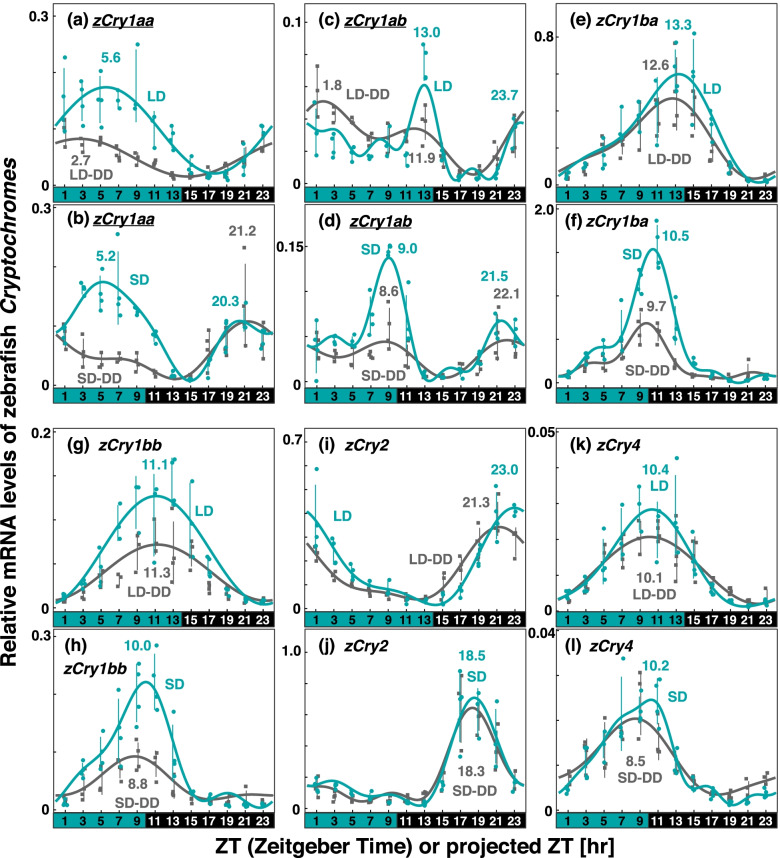
Comparison of *Cry* expression profiles in zebrafish eyes under various light conditions. Eyeballs (*n* = 3–4) were collected every 2 h from zebrafish entrained under turquoise green light on long-days or short-days during (LD or SD, green dots) or on the first day in DD after LD or SD entrainment (LD-DD or SD-DD, gray squares). Expression levels of each mRNA were calculated relative to the synergistic mean of *zEf1α* and *zGapdh* expression levels. Error bars indicate standard deviation. The expression profiles approximated by the cosinor fitting are indicated (LD or SD, green curves; LD-DD or SD-DD, gray curves) with the estimated peak time. The results of Kruskal–Wallis test and Dann-Bonferroni post-hoc test are shown in Table S3 and Figs S2-S5, respectively. *Cry* genes showing significant change (*p* < 0.05, Mann–Whitney U test) in the averaged mRNA levels between LD/SD and LD-DD/SD-DD are underlined. Light and dark conditions are indicated at the bottom of each panel

To compare the photoperiodic response of each *Cry* gene in the zebrafish eye, we measured their expression patterns under the LD and SD conditions (green curves and plots in Fig. 2; Table 1, Figs. S2 and S3). We also investigated the *zCry* expression patterns on the first day in DD after the entrainment under LD or SD condition to discriminate between regulations by external photoperiod and photoperiod-dependent internal circadian signals (LD-DD and SD-DD, gray curves and plots in Fig. 2; Table 2, Figs S4 and S5). The peak times were estimated using the cosinor fitting (Tables 1 and 2) against ZT (time since the beginning of the light period; for the peak times in LD and SD) or projected ZT (pZT, time since the projected beginning of the light period; for those in LD-DD and SD-DD).

**Table 1:** Peak times, responses to photoperiod, and mode of synchronization of *Cry* genes in LD and SD

Gene	Peak time in ZT^a^		Peak time in MNFT^b^		Peak time in SSFT^c^		Phase Difference (hr) between LD and SD			Mode of Synchronization
	LD	SD	LD	SD	LD	SD	ΔZT	ΔMNFT^b^	ΔSSFT^c^	
Zebrafish										
* zCry1aa*	5.60	5.19	10.60	12.19	15.60	19.19	**0.41**	1.59	3.59	Sunrise
* zCry1aa_2nd*	(5.60)	20.31	(10.60)	3.31	(15.60)	10.31	9.29	7.29	**5.29**	Sunset
* zCry1ab_M*	23.66	21.51	4.66	4.51	9.66	11.51	2.15	**0.15**	1.85	Midnight/Midday
* zCry1ab_E*	13.00	9.03	18.00	16.03	23.00	23.03	3.97	1.97	**0.03**	Sunset
* zCry1ba*	13.31	10.46	18.31	17.46	23.31	0.46	2.85	**0.85**	1.15	Midnight/Midday
* zCry1bb*	11.12	9.98	16.12	16.98	21.12	23.98	1.14	**0.86**	2.86	Midnight/Midday
* zCry2*	22.97	18.49	3.97	1.49	8.97	8.49	4.48	2.48	**0.48**	Sunset
* zCry4*	10.38	10.23	15.38	17.23	20.38	0.23	**0.15**	1.85	3.85	Sunrise
Goldfish										
* gCry1aa*	5.04	4.06	10.04	11.06	15.04	18.06	**0.98**	1.02	3.02	Sunrise
* gCry1ab*	12.61	10.14	17.61	17.14	22.61	0.14	2.47	**0.47**	1.53	Midnight/Midday
* gCry1ba*	13.06	11.05	18.06	18.05	23.06	1.05	2.01	**0.01**	1.99	Midnight/Midday
* gCry1bb*	10.81	9.44	15.81	16.44	20.81	23.44	1.37	**0.63**	2.63	Midnight/Midday
* gCry2*	22.02	19.59	3.02	2.59	8.02	9.59	2.43	**0.43**	1.57	Midnight/Midday
* gCry4*	13.18	12.47	18.18	19.47	23.18	2.47	**0.71**	1.29	3.29	Sunrise
Medaka										
* OlCry1aa*	3.85	4.77	8.85	11.77	13.85	18.77	**0.92**	2.92	4.92	Sunrise
* OlCry1ab*	2.68	1.37	7.68	8.37	12.68	15.37	1.31	**0.69**	2.69	Midnight/Midday
* OlCry1ba*	15.42	9.17	20.42	16.17	1.42	23.17	6.25	4.25	**2.25**	Sunset
* OlCry2*	4.08	1.94	9.08	8.94	14.08	15.94	2.14	**0.14**	1.86	Midnight/Midday

^a^ Zeitgeber time

^b^ Midnight-fitting time

^c^ Sunset-fitting time

**Table 2:** Peak times, responses to photoperiod, and mode of synchronization of *Cry* genes in DD after LD and SD

Gene	Peak time in pZT^a^		Peak time in pMNFT^b^		Peak time in pSSFT^c^		Phase Differences (hr) between LD-DD & SD-DD			Mode of Synchronization
	LD-DD	SD-DD	LD-DD	SD-DD	LD-DD	SD-DD	ΔpZT	ΔpMNFT^b^	ΔpSSFT^c^	
Zebrafish										
* zCry1aa*	2.67	21.24	7.67	4.24	12.67	11.24	5.43	3.43	**1.43**	Sunset
* zCry1ab_M*	1.80	22.12	6.80	5.12	11.80	12.12	3.68	1.68	**0.32**	Sunset
* zCry1ab_E*	11.86	8.58	16.86	15.58	21.86	22.58	3.28	1.28	**0.72**	Sunset
* zCry1ba*	12.61	9.74	17.61	16.74	22.61	23.74	2.87	**0.87**	1.13	Midnight/Midday
* zCry1bb*	11.29	8.76	16.29	15.76	21.29	22.76	2.53	**0.53**	1.47	Midnight/Midday
* zCry2*	21.31	18.28	2.31	1.28	7.31	8.28	3.03	1.03	**0.97**	Sunset
* zCry4*	10.12	8.49	15.12	15.49	20.12	22.49	1.63	**0.37**	2.37	Midnight/Midday
Goldfish										
* gCry1aa*	2.67	22.74	7.67	5.74	12.67	12.74	3.93	1.93	**0.07**	Sunset
* gCry1ab*	13.47	10.58	18.47	17.58	23.47	0.58	2.89	**0.89**	1.11	Midnight/Midday
* gCry1ba*	12.59	10.08	17.59	17.08	22.59	0.08	2.51	**0.51**	1.49	Midnight/Midday
* gCry1bb*	12.42	9.16	17.42	16.16	22.42	23.16	3.26	1.26	**0.74**	Sunset
* gCry2*	23.08	21.00	4.08	4.00	9.08	11.00	2.08	**0.08**	1.92	Midnight/Midday
* gCry4*	14.69	11.95	19.69	18.95	0.69	1.95	2.74	**0.74**	1.26	Midnight/Midday
Medaka										
* OlCry1aa*	23.49	22.27	4.49	5.27	9.49	12.27	1.22	**0.78**	2.78	Midnight/Midday
* OlCry1ab*	1.74	1.00	6.74	8.00	11.74	15.00	**0.74**	1.26	3.26	Sunrise
* OlCry1ba*	11.68	10.99	16.68	17.99	21.68	0.99	**0.69**	1.31	3.31	Sunrise
* OlCry2*	23.12	20.47	4.12	3.47	9.12	10.47	2.65	**0.65**	1.35	Midnight/Midday

^a^ Projected Zeitgeber time

^b^ Projected midnight-fitting time

^c^ Projected sunset-fitting time

The levels of *zCry1aa* mRNA were high in the morning and around noon in the light period (ZT5.6 in LD; ZT5.2 in SD) under both LD and SD conditions (Fig. 2a and b). In SD, a second peak appeared in the second half of the night (ZT20.3) and the similar peak was also observed in DD after SD (pZT21.2 in SD-DD, Fig. 2b). Under both LD-DD and SD-DD conditions, a peak around noon (ZT5.6 in LD; ZT5.2 in SD) weakened or disappeared (Fig. 2a and b). Averaged levels of *zCry1aa* mRNA in LD and SD were significantly higher than those in LD-DD and SD-DD, respectively (underlines in Fig. 2a and Fig. b, Table S6, Mann–Whitney U test).

*zCry1ab*, as previously reported [22], showed two peaks near the beginning of the light period (ZT23.7 in LD; ZT21.5 in SD) and just before the end (ZT13.0 in LD; ZT9.0 in SD), under both LD and SD conditions (Fig. 2c and d; Table 1). In both LD-DD and SD-DD, these morning and evening peaks were observed (Fig. 2c and d; Table 2), but the evening peaks (pZT11.9 and pZT8.6) were likely blunted. Averaged levels of *zCry1ab* mRNA in LD and SD were significantly higher than those in LD-DD and SD-DD, respectively (underlines in Fig. 2c and Fig. d, Table S6, Mann–Whitney U test).

*zCry1ba/1bb/4* all had a peak from the late light period to early dark period (ZT10.4–13.3 in LD; ZT10.0–10.5 in SD), regardless of the day length (Fig. 2e–h, k and l). Similar profiles were observed also undr both the LD-DD and SD-DD conditions.

*zCry2* showed a peak from the end of the dark period to the beginning of the light period in LD (ZT23.0, Fig. 2i) but shifted to the middle of the dark period in SD (ZT18.5, Fig. 2j). Peaks similar to this peak were observed also in LD-DD (pZT21.3, Fig. 2i) and SD-DD (pZT18.3, Fig. 2j).

### Daily and circadian variations in the expression of goldfish and medaka *Cry*

Next, we measured the expression patterns of *Cry* genes in the goldfish eye and medaka eye under LD/SD (Figs. 3 and 4; Table 1, Figs. S6, S7, S10, and S11) and LD-DD/SD-DD (Figs. 3 and 4; Table 2, Figs. S8, S9, S12, and S13) conditions.

**Fig. 3:**
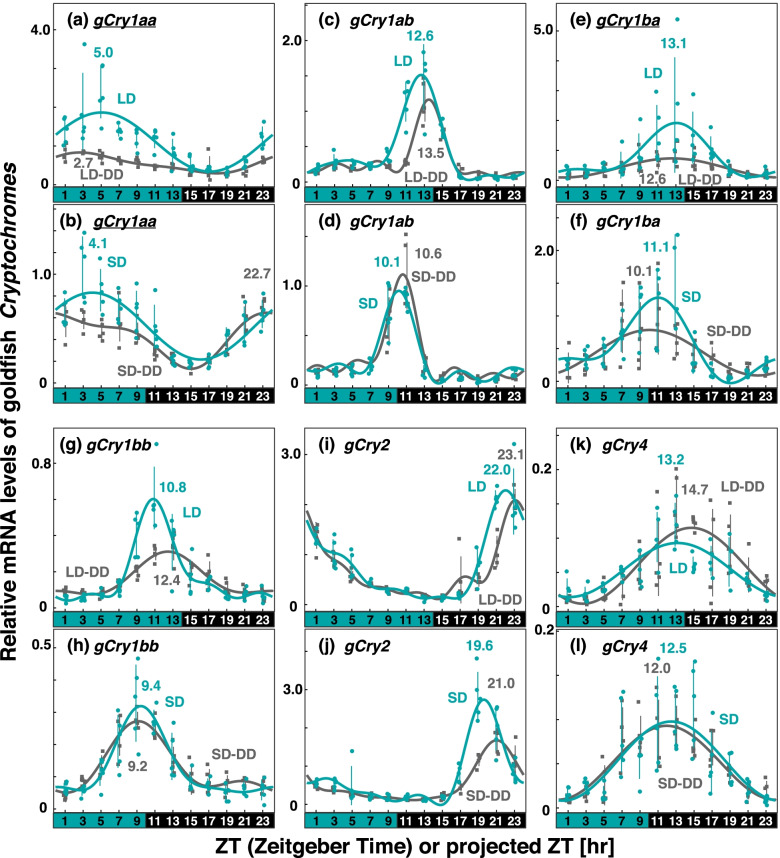
Comparison of *Cry* expression profiles in goldfish eyes under various light conditions. Eyeballs (*n* = 4–5) were collected every 2 h from goldfish entrained under turquoise green light on long-days or short-days during (LD or SD, green dots) or on the first day in DD after LD or SD entrainment (LD-DD or SD-DD, gray squares). Expression levels of each mRNA were calculated relative to the synergistic mean of *gGusb, gPgk1,* and *gHprt1* expression levels. Error bars indicate standard deviation. The expression profiles approximated by the cosinor fitting are indicated (LD or SD, green curves; LD-DD or SD-DD, gray curves) with the estimated peak time. The results of Kruskal–Wallis test and Dann-Bonferroni post-hoc test are shown in Table S4 and Figs S6–S9, respectively. *Cry* genes showing significant change (*p* < 0.05, Mann–Whitney U test) in the averaged mRNA levels between LD/SD and LD-DD/SD-DD are underlined. Light and dark conditions are indicated at the bottom of each panel

**Fig. 4:**
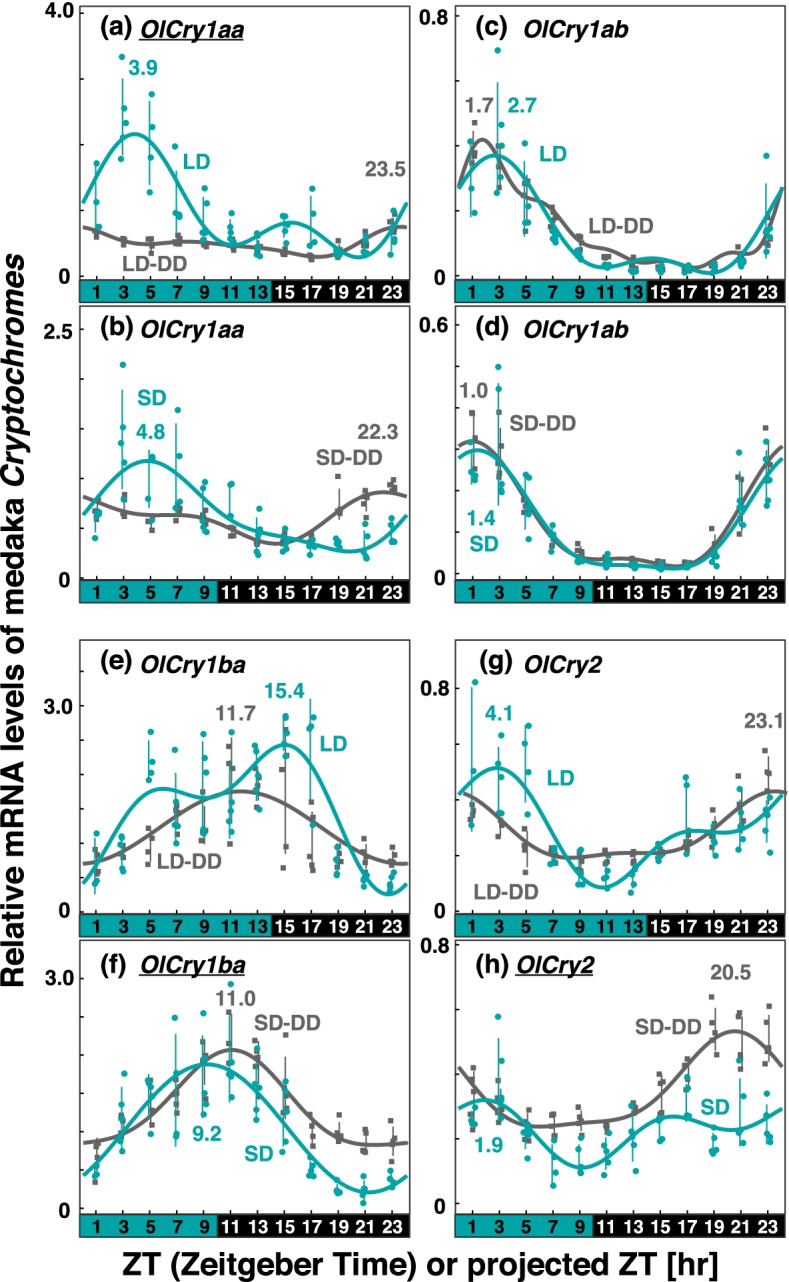
Comparison of *Cry* expression profiles in medaka eyes under various light conditions. Eyeballs (*n* = 3–5) were collected every 2 h from medaka entrained under turquoise green light on long-days or short-days during (LD or SD, green dots) or on the first day in DD after LD or SD entrainment (LD-DD or SD-DD, gray squares). Expression levels of each mRNA were calculated relative to the synergistic mean of *OlGusb, OlEf1α*, and *OlHprt1* expression levels. Error bars indicate standard deviation. The expression profiles approximated by the cosinor fitting are indicated (LD or SD, green curves; LD-DD or SD-DD, gray curves) with the estimated peak time. The results of Kruskal–Wallis test and Dann-Bonferroni post-hoc test are shown in Table S5 and Figs S10–S13, respectively. *Cry* genes showing significant change (*p* < 0.05, Mann–Whitney U test) in the averaged mRNA levels between LD/SD and LD-DD/SD-DD are underlined. Light and dark conditions are indicated at the bottom of each panel

*gCry1aa* showed expression profiles with a peak in the first half of the light period (ZT5.0 in LD; ZT4.1 in SD) in both LD and SD, like *zCry1aa* (green curves and plots in Fig. 3a and b; Table 1). *gCry1aa* showed weaker but significant oscillations also in DD (gray curves and plots in Fig. 3a and b). Averaged levels of *gCry1aa* mRNA in LD and SD were significantly higher than those in LD-DD and SD-DD, respectively (underlines in Fig. 3a and Fig. b). *gCry1ab* did not show dual peaks like *zCry1ab*, but showed a peak only around the beginning of the dark period (ZT12.6 in LD; ZT10.1 in SD) and the projected beginning of the dark period of the entrainment cycle (pZT13.5 in LD-DD; pZT10.6 in SD-DD; Fig. 3c and Fig. d). The shift widths of the peaks in LD and SD were different (see below); however, *gCry1ba/1bb/2/4* (Fig. 3e-3l) showed daily variations in the patterns, like that in *zCry1ba/1bb/2/4*. These patterns were mostly conserved in LD-DD and SD-DD except for *gCry1ba* in LD (Fig. 3e); the averaged level of *gCry1ba* mRNA in LD was significantly higher than that in LD-DD (underline in Fig. 3e).

*OlCry1aa*, like *Cry1aa* in the other two fish species, showed a light-dependent peak in the first half of the light period (ZT3.9 in LD; ZT4.8 in SD; Fig. 4a and b, Table 1), while its averaged mRNA level was significantly upregulated only when entrained under LD condition (underline in Fig. 4a). Additionally, a peak was observed in the late subjective night in LD-DD (pZT23.5 in LD-DD, Fig. 4a) and SD-DD (pZT22.3, Fig. 4b). The expression pattern of *OlCry1ab* (Fig. 4c and d), on the other hand, was different from that of *zCry1ab* and *gCry1ab*, and showed a peak in the morning (ZT2.7 in LD; ZT1.4 in SD) and early subjective day (pZT1.7 in LD-DD; pZT1.0 in SD-DD). *OlCry1ba* (Fig. 4e and f) exhibited a similar pattern to that of *zCry1ba* (Fig. 2e and f) and *gCry1ba* (Fig. 3e and f). *OlCry2* (Fig. 4g), unlike the *Cry2* in the other fish, had a peak in the morning (ZT4.1 in LD; ZT1.9 in SD). In DD, *OlCry2* had a peak in the latter half of the subjective night (pZT23.1 in LD-DD, Fig. 4g; pZT20.5 in SD-DD, Fig. 4h), and the averaged mRNA level in SD-DD was significantly higher than that in SD (underline in Fig. 4h).

### Evaluation of the mRNA expression peaks of *Cry*

**Fig. 5:**
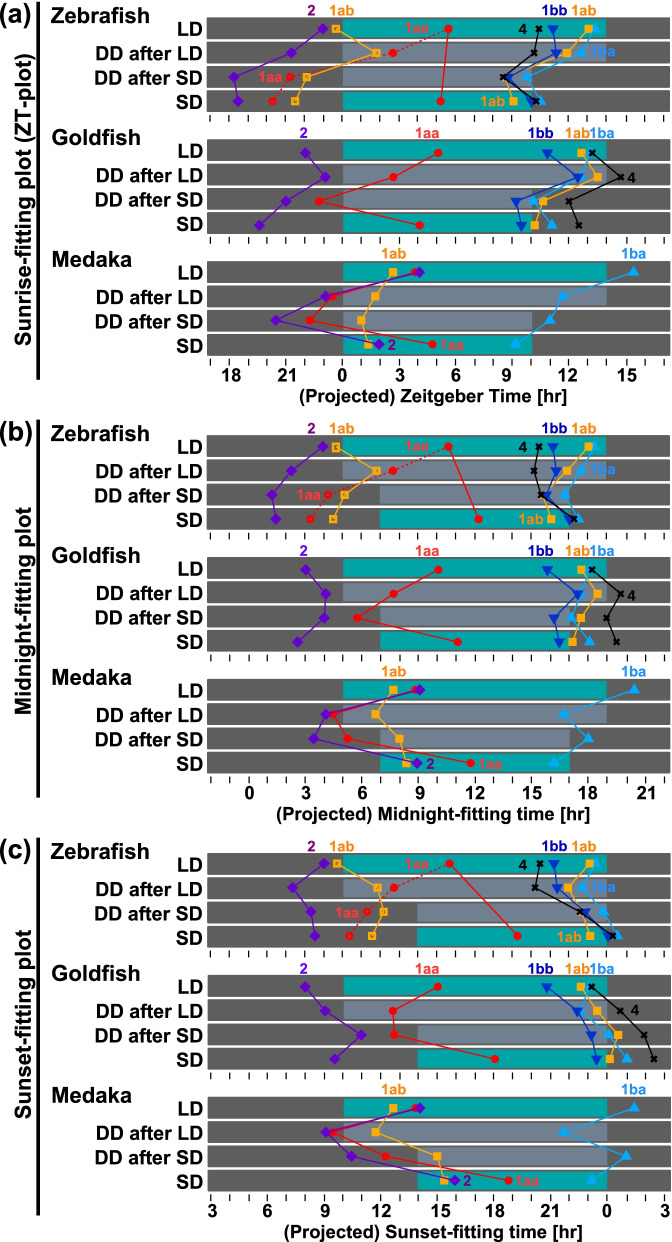
Phase shifts of *Cry* expression under various light conditions. The peak time (acrophase) obtained from the fitted curve by the cosinor fitting is plotted against the time elapsed from the beginning of the light period (panel a, sunrise-fitting plot), the middle of the night (panel b, midnight-fitting plot), and the beginning of the dark period (panel c, sunset-fitting plot). The morning peak of *zCry1ab* is indicated as yellow open squares, and the evening peak is shown using yellow closed squares. Blue/dark bars indicate light conditions and gray bars indicate the subjective night period. LD, long-day; SD, short-day; DD after LD, constant dark just after the long-day entrainment (LD-DD in the text); DD after SD, constant dark just after the short-day entrainment (SD-DD in the text)

**Fig. 6:**
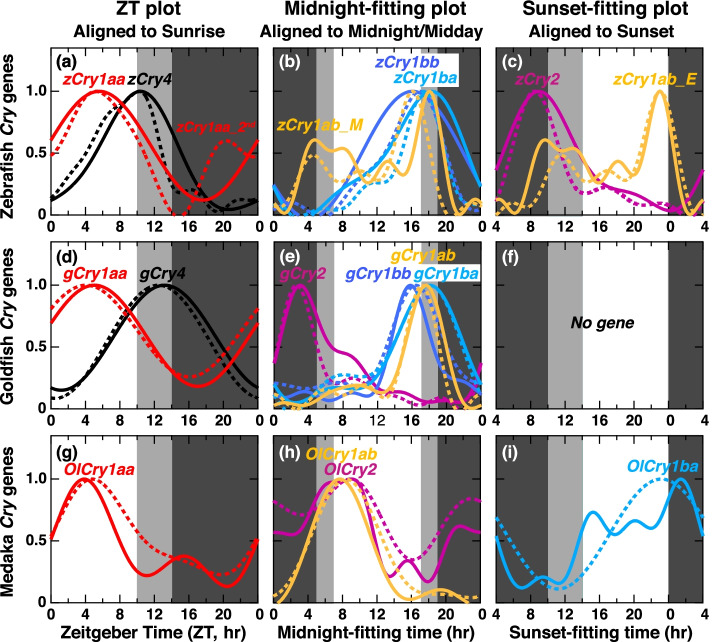
Comparison of expression profiles of *Cry* genes in LD or SD aligned to sunrise, midnight, or sunset. **a**, **d**, **g** Expression profiles of the genes with the minimum shifts in the Zeitgeber Time (ZT) plot. **b**, **e**, **h** Expression profiles of the genes with the minimum shifts in the midnight-fitting plot (hours after midnight). **c**, **i** Expression profiles of the genes with the minimum shifts in the sunset-fitting plot (hours after sunset). There was no gene for sunset-fitting plot in goldfish (**f**). The expression profiles approximated using the cosinor fitting in LD and SD are shown in normal curves and dotted curves, respectively

**Fig. 7:**
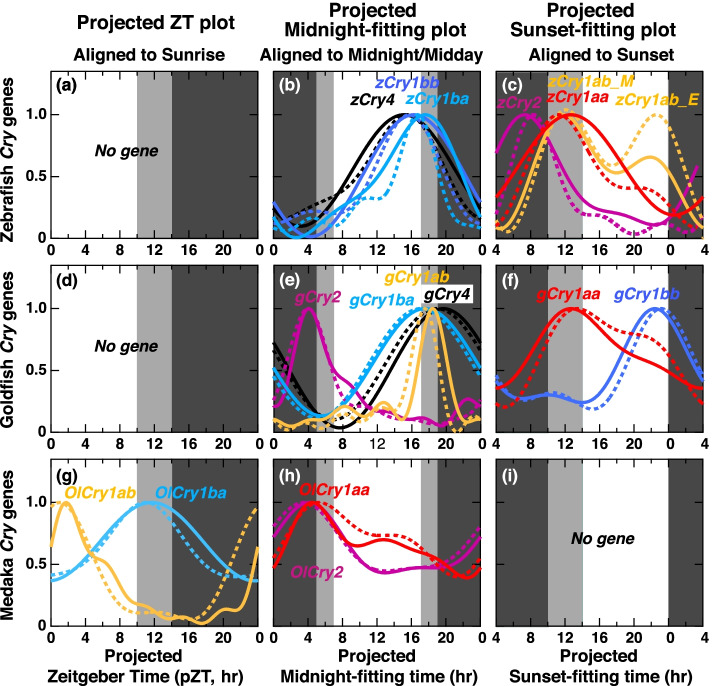
Comparison of expression profiles of *Cry* genes in the dark after entrainment under LD (LD-DD) or SD (SD-DD) aligned to projected sunrise, midnight, or sunset. **g** Expression profiles of the genes with the minimum shifts in the projected Zeitgeber Time (pZT) plot. **b**, **e**, **h** Expression profiles of the genes with the minimum shifts in the projected midnight-fitting plot. **c**, **f** Expression profiles of the genes with the minimum shifts in the projected sunset-fitting plot. There was no gene for pZT plot in zebrafish (**a**) or goldfish (**d**) or projected sunset-fitting plot in medaka (**i**). The expression profiles approximated using the cosinor fitting in LD-DD and SD-DD are shown in normal curves and dotted curves, respectively

For evaluating the expression profile of each *Cry* gene in response to the changes in day length, we plotted the peak times against ZT or pZT (Fig. 5a). We found that the phase of *zCry1aa* and *zCry4* did not change largely between LD and SD, while that of *zCry2* and *OlCry1ba* exhibited large shifts. To understand the synchronization mode of each gene, we recalculated and plotted the peak times with using midnight as the reference point ([projected] midnight-fitting plot; Fig. 5b, Table 1, peak time in midnight-fitting time [MNFT] or projected MNFT [pMNFT]), which is the time elapsed since midnight. Similarly, we also recalculated and plotted the peak times with the time elapsed since sunset (Fig. 5c, Table 1, sunset-fitting time [SSFT]) or projected SSFT [pSSFT]. For each gene, we further compared values of the shift width between LD and SD (ΔZT, ΔMNFT, ΔSSFT in Table 1) or between LD-DD and SD-DD(ΔpZT, ΔpMNFT, ΔpSSFT in Table 2). Then, we classified the peak using the plot with the minimum shift (boldfaced in phase differences in Tables 1 and 2) into 3 groups possibly corresponding to the 3 modes of synchronization, “Sunrise”, “Midnight/Midday”, and “Sunset”. Then, we compared the shape of the expression profiles of each *Cry* gene (Figs. 2–4) by overlaying them to ascertain the smallest phase differences in LD vs SD (Fig. 6, Table 1) or LD-DD vs SD-DD (Fig. 7, Table 2).

In LD and SD, *Cry1aa* and *Cry4* showed the least shift widths in the ZT plot (Figs. 5a, 6d, and g), while the morning peak of *zCry1ab* (*zCry1ab_M*), and peaks of *zCry1ba*, *zCry1bb*, *gCry1ab*, *gCry1ba*, *gCry1bb*, *gCry2*, *OlCry1ab*, and *OlCry2* showed the least shift widths in the midnight-fitting plot (Figs. 5b, 6b, e, and h). The evening peak of *zCry1ab* (*zCry1ab_E*), and peaks of *zCry2*, and *OlCry1ba* showed the least shift widths in the sunset-fitting plot (Figs. 5c, 6c, and i, Table 1). The profiles of *Cry* expressions in LD and SD (Fig. 6) not only showed conserved peak times but also matched with each other, except for those of *zCry1aa* (Fig. 6a) and *zCry1ab* (Fig. 6b). The profiles in LD and SD of *zCry1aa* and *zCry1ab*, which have dual peaks, did not match with each other regarding their photoperiod dependency.

In LD-DD and SD-DD, peaks of *OlCry1ab* and *OlCry1ba* showed the least shift widths in the pZT plot (Figs. 5a and 7g), but all peaks of the other *Cry* genes showed the least shift widths in the projected midnight-fitting plot (Figs. 5b, 7b, e, and h; Table 2) or projected sunset-fitting plot (Figs. 5c, 7c, and f; Table 2). The profiles in LD-DD and SD-DD were mostly similar to each other (Fig. 7), and the synchronization mode of many peaks changed from those in LD and SD (Fig. 6): Peaks of *Cry1aa* and *Cry4* showed sunrise synchronization mode in LD and SD (Fig. 6a, d, and g) but changed to the other modes in LD-DD and SD-DD (Fig. 7b, c, e, f, and h). Peaks of *gCry1bb*, *OlCry1ab*, and *OlCry1ba* also changed the synchronization mode (Figs. 6 and 7).

## Discussion

### *Cry* genes in goldfish and medaka

In this study, we analyzed the *Cry* gene expression in zebrafish, which lives in the subtropics, and in goldfish and medaka, which live in the temperate zone. Among these three species, zebrafish and goldfish, which are evolutionarily close to each other, had the same set of six *Cry* genes (*Cry1aa/1ab/1ba/1bb/2/4*); while in medaka, *Cry4* and *Cry1bb* are absent and are considered to have been lost. CRY4 is a possible geomagnetic receptor [13–16]; therefore, the lack of *Cry4* may be relevant to the habitat of medaka, which possibly localizes relying on vision regardless of magnetic sensation. Alternatively, another mechanism mediated by the other CRYs or a magnetite-based system [31] may operate in medaka. Zebrafish and goldfish had the same set of *Cry* genes, with almost similar expression profiles (exception: *Cry1ab*). The expression profiles of *Cry*s in medaka were immensely different from those in zebrafish and goldfish (Fig. 5). This diversity may be due to differences in habitat or because of the evolutionary separation of medaka.

### *zCry1aa* as well as *zCry1ab* showed dual peaks

We previously compared the expression profiles of the *Cry* genes in the zebrafish eye and reported that their peak times could be located on midnight-morning and evening periods, and that *Cry1ab* has a double peak [22]. In this study, expression profiles of 16 *Cry* genes were obtained by evaluating the gene expression every 2 h, under different photoperiods (14L10D and 10L14D) and in the constant dark just after the different photoperiodic entrainments.

Genes showing sunrise-synchronized peaks in zebrafish and goldfish seem to be influenced by light. One of such genes, *zCry1aa*, is strongly induced by light in both zebrafish tissues [22] and cultured cell lines [32, 33]. This seemed to be the case also in the zebrafish eye under both the SD and LD conditions, because a peak around noon weakened or disappeared under both LD-DD and SD-DD conditions (Fig. 2a and b). Interestingly, *zCry1aa* showed a second peak in the latter half of the night in SD (ZT20.31; *zCry1aa_2nd* in Table 1), which remained in SD-DD (pZT21.24). Thus, the mRNA expression profile of *zCry1aa* dynamically changed in a photoperiod-dependent manner, and it is likely driven by both external light and internal oscillator simultaneously. Such photoperiodic expressions of *zCry1aa* and *zCry1ab* may contribute to the PTM in zebrafish. In goldfish and medaka, *Cry* genes showed no clear double peak; however, their peak phases and responsiveness to different photoperiods were diverged, implying their role in the PTM (see below).

### Classification of *Cry* expression peaks based on their photic and photoperiodic responses

We further compared the expression peaks of each *Cry* gene, and calculated the difference using three types of plots, ZT (Zeitgeber time), midnight-fitting (MNFT), and sunset-fitting (SSFT), with each using the sunrise (light onset) or midnight/midday or sunset (light offset) as the reference point (Fig. 5, Table 1). Based on the magnitudes of the phase shift in the three types of calculation, we classified the peak into three modes: Sunrise-, midnight/midday-, and sunset-synchronized modes (Tables 1 and 2), which likely better synchronize to light-on, midpoints of light and dark periods, and light-off, respectively. These three modes may possibly correspond to multiple oscillators having distinct reference points. Such oscillators may differentially respond to external light–dark cycles and photoperiods.

Notably, six peaks (*zCry1ba/bb*, *gCry1ab/1ba/2*, *OlCry2*) were classified into the midnight/midday-synchronized modes under all the examined light conditions (LD, SD, LD-DD, and SD-DD conditions): Peak phases of *zCry1ba/bb* and *gCry1ab/1ba* located around the midpoint from midday to midnight (Fig. 6b and f, underlines in Tables 1 and 2), suggesting the presence of midnight/midday-synchronized circadian clocks served by these *Cry* genes in zebrafish and goldfish. In these fish species, no sunrise-synchronized peak was observed under constant dark condition (Table 2), implying that sunrise-synchronized peaks of *zCry1aa/4* and *gCry1aa/4* were due not to sunrise-synchronized clock but to light-dependent upregulation. Thus, zebrafish and goldfish seem to retain at least two common sets of circadian oscillators, midnight/midday-synchronized and sunset-synchronized oscillators, to which light signal may input to modulate the expression profiles to form the sunrise-synchronized peaks.

In medaka, peaks and profiles of *OlCry1ab* and *OlCry1ba* showed the synchronization of sunrise mode under LD-DD and SD-DD conditions (Fig. 7g), while no gene showed sunset mode. Both the phase and synchronization mode of the *Cry* expression peaks in medaka are different from those in zebrafish and goldfish (Fig. 7), suggesting a different set of circadian clocks in the medaka eye, namely, sunrise-synchronized and midnight/midday-synchronized oscillators (Table 2).

We did not statistically evaluate the photic regulation of each peak of the *Cry* genes, because larger numbers of samples would be necessary for more precise comparisons of the expression levels under different light conditions at each peak time point. However, comparisons of *Cry* expression profiles (Figs. 2–4) imply that some peaks may be ascribed to photic regulation. As well as *zCry1aa/4* and *gCry1aa/4* discussed above, *zCry1ab* (evening peak), *zCry1bb*, *gCry1ba*, *OlCry1aa*, and *OlCry2* seem to be regulated by light. It would be important to know how these genes regulate the transcription of circadian genes constituting each of the three oscillators having distinctive modes of synchronization.

### Photoperiodic time measurement

An internal and an external coincidence model have been proposed as major models for the PTM [5, 6, 34]. In the internal coincidence model, the photoperiodic reaction is induced by the phase relationships of the internal oscillators, in which the steady states of multiple oscillators change depending on the photoperiod. In the external coincidence model, the photoperiodic reaction is induced when a light stimulus occurs within the specific time period in a day, called the photo-inducible phase. As assumed by this model, the photoperiod can also be determined by a combination of the internal clock signal and the external light signal.

Considering the present results, the multiple oscillators in the internal coincidence model may possibly correspond to the circadian clocks synchronized to the sunrise, midnight/midday, and sunset, phase relationships of which would change in response to the photoperiod. All three fish species examined in this study likely retain at least two clocks showing different responses to photoperiods in *Cry* mRNA profiles (Tables 1 and 2). Therefore, it seems possible to detect the photoperiod according to the internal coincidence model by a combination of the time signals from the two clocks.

On the other hand, the external coincidence model would still be possible by using photoresponsive circadian genes such as *zCry1aa*. This is because the light period starts at different phases of the internal sunset-synchronized and midnight/midday-synchronized oscillators depending on the photoperiod. In fact, *zCry1aa* showed two peaks in the light and dark periods only under the SD condition, which may result in larger day/night variation in LD than SD. Future examination of the integration of the temporal signals from the circadian oscillators and the photosignals at the level of transcription regulation may help to determine which model fits better to the possible PTM in the fish eye.

In mammals, the photosignals from the eye are transmitted to the pineal gland and the day length information regulates the plasma melatonin levels, which in turn regulates the photoperiodic response in the pituitary gland [35, 36]. In quail, the photoperiodic center for time measurement and the photoreceptor cells are localized in the median eminence and the paraventricular organ in the deep part of the brain, respectively [37, 38]. In fish, the saccus vasculosus is the central site for PTM [39]. Despite this accumulating knowledge, the molecular mechanism underlying the PTM is not known in any species. The eye is simpler than the brain network or other complex regulatory circuits in the whole body, and therefore, the photoperiodic eye may be a suitable model for elucidating the molecular mechanism underlying the PTM.

One of the important issues to be addressed in future studies is whether the midnight/midday-synchronized oscillator and the sunset-synchronized oscillator are localized within a single cell. This can be investigated by establishing retina-derived cell lines, although non-retinal cell lines, such as Z3 [32] and PAC2 [33], have been routinely used in chronobiology. Fugu Eye cells [40] are an eye-derived cell line and we have reported the photic response and autonomous oscillation of clock genes in these cells [41]. It would be interesting to investigate the photoperiodic expression of clock gene in Fugu Eye cells.

### Midnight/midday-synchronized clock for sun compass

The midnight/midday-synchronized clock might also be of physiological importance for sun-compass orientation, which tells the animals the geographic direction determined from the time signal and the position of the sun [42]. In insects, the circadian clock is essential for a sun compass [43], and it would be interesting to speculate that the midnight/midday-synchronized clock may enable the organisms to acquire the time signal relative to noon. Such an information would seem to help maintain the accurate function of a sun compass especially in mid-high latitude regions where there are seasonal day-length changes. A sun compass has been shown in coral reef fish larvae [44] and Mediterranean fish larvae [45]. Polarization vision, which may help in ascertaining the position of the sun by detecting skylight polarization, was reported in goldfish [46], although the existence of a sun compass has not been reported in the three fish species used in this study. A combination of polarization vision and a midnight/midday-synchronized clock in the eye might, therefore, possibly constitute the sun compass in goldfish.

## Conclusions

In this study, we showed that the eyes of the three fish species retain a midnight/midday-synchronized circadian clock, which may play pivotal roles in the detection of daylength. Although the present observations may not provide a complete answer to the mechanism of the PTM, they would provide important clues to uncover the PTM mechanism. In the future, genome-wide expression analyses, evaluation of the regulatory regions of *Cry* genes, characterizing interactions of CRYs with other clock proteins, and understanding the physiological significance of the midnight/midday-synchronized clock, would provide valuable insights.

## Methods

### Ethics statement

All experiments were conducted in accordance with the guidelines and regulations of Waseda University. All protocols were approved by the Committee for the Management of Biological Experiments at Waseda University, and experimental animal care was conducted with permission from the Animal Experiment Committee of Waseda University (approval number: 2019-A039, 2020-A118, 2021-A031).

### Animals

Zebrafish (*Danio rerio*), goldfish (*Carassius auratus*), and medaka (*Oryzias latipes*) were obtained from a local supplier and maintained in tanks (6.5 cm × 25 cm, 14 cm water depth) under long-day conditions (14 h light: 10 h dark; lights on at 9:00 am; 5–20 μW cm^−2^, fluorescent light, FHF32EX-N-HX-S, NEC). They were fed twice per day with living baby brine shrimp or commercial pellets. The temperature of the circulating water was kept at 26–28 °C (zebrafish), 27–28 °C (goldfish), and 25–26 °C (medaka).

### Primers

Primers used for zebrafish were the same as those used in a previous study [22]. Using Primer3 (ver. 0.4.0, http://bioinfo.ut.ee/primer3-0.4.0/), primer sets for amplification of medaka and goldfish eye cDNA (Tables 3 and 4) were designed based on the genome databases (Goldfish [ASM336829v1], https://asia.ensembl.org/Carassius_auratus/Info/Index; Japanese medaka HdrR [ASM223467v1], https://asia.ensembl.org/Oryzias_latipes/Info/Index). They were designed to sandwich an intron in the amplification region, and more than two primer sets were examined for one gene. When we found multiple transcripts originated from a single gene and/or highly conserved genes due to a recent gene duplication, primers were designed to cover common sequences as possible. Serially diluted eye cDNA was subjected to quantitative RT-PCR for selecting primers with amplification efficiency close to 100%. Primers were selected based on the amplification curve, shape of the melting curve, and the amplification efficiency. The PCR products were cleaned with Gen Elute PCR Clean-Up Kit (SIGMA, NA1020) and subjected to direct sequencing by Eurofins DNA sequence service (https://eurofinsgenomics.jp/jp/home.aspx) to confirm the identities of the amplified cDNA. In addition, quantitative RT-PCR on eye samples of various ZTs (ZT1, ZT7, ZT15, and ZT21 for goldfish; ZT0, ZT6, ZT12, and ZT18 for medaka) was used for selecting the control genes from 8 (goldfish) and 7 (medaka) candidate genes. The control genes with the least temporal fluctuation of expression were selected (Tables 3 and 4).

**Table 3:** Primers used in qPCR of goldfish *Cry* genes

Gene	Primer	Sequence (5’ to 3’)	Amp. Eff. (%)
*gCry1aa*	Forward	TGGCC TGGAG GAGAA ACAGA	94.2
	Reverse	CAACA GCGAA TTGGC ATTCA	
*gCry1ab*	Forward	CAGTG TCATG TGGTC AACTG TCTG	101.0
	Reverse	TGTCC CTCTCC CTCTC TGTGA	
*gCry1ba*	Forward	TTTAC CGCGG GAAGA GATGA	90.6
	Reverse	TGCAG GGTTG TCATG GAGAC	
*gCry1bb*	Forward	CGCCT GAACA TCGAG AGGAT	94.3
	Reverse	CCCAC GACCT CCATG TGATT	
*gCry2*	Forward	GGCAC AGGAA TATGG TGTGG A	95.2
	Reverse	GAGTC GATTC ACAAT GGCTT GA	
*gCry4*	Forward	CGCAT TCTTC CACAA ATACA CC	95.0
	Reverse	ACGTC TTCTG GAGCT TTCCA C	
*gGusb*	Forward	ACCGG GAACC ATCCA GTACA	96.3
	Reverse	CGATC GGTGT ATTCC AGCGT A	
*gPgk1*	Forward	CGTTG GACAA GGTGG ATGTG	101.7
	Reverse	TGATG GAACT GCAGC CTTGA	
*gHprt1*	Forward	AAGTG GCCAG TTTGC TGGTG	104.5
	Reverse	GTCAA GTGCA TATCC AACCA CA

**Table 4:** Primers used in qPCR of medaka *Cry* genes

Gene	Primer	Sequence (5’ to 3’)	Amp. Eff. (%)
*OlCry1aa*	Forward	TGTCC TGCCG CCTCT TTTAC	108.5
	Reverse	AAAAG AATTC ACGCC ACAGC AG	
*OlCry1ab*	Forward	ACCCT GCGCT GCATC TAC	113.2
	Reverse	CCTCG GATGA CGAAC AAGC	
*OlCry1ba*	Forward	CAACA TCGAG AGGAT GAAGC AG	91.4
	Reverse	TCCTC CATTC CCGTT CCCAT	
*OlCry2*	Forward	TATTC TGGAC CCCTG GTTCG	102.8
	Reverse	CCTGG AGTTG AGCTT CTTCA G	
*OlEf1α*	Forward	TCTAC AAGTG CGGAG GAATC G	94.7
	Reverse	GTCCA ACACC CAGGC GTACT	
*OlHprt1*	Forward	ACCGC TCCAT TCCCA TGAC	98.0
	Reverse	TGCCG GTTAG GGTAG ACAGG	
*OlGusb*	Forward	TGACC CACGA GAATC CAGGT	99.3
	Reverse	CGGAT GCCAA CAGGA AGAGT	

### Sampling

We entrained the fishes in the stock tanks under LD or SD conditions for at least 7 days. The fishes were then transferred into an incubator set at 26 °C (zebrafish) or 28 °C (goldfish) or 25 °C (medaka) and entrained under turquoise green LED light (3 W Turquoise Green LED, EPILED, Future Eden; Fig. S1; λmax = 500 nm; λ_1/2_ = 488 nm and 514 nm; 210–240 μW cm^−2^ near the surface of the water) for 4 days. We used blue light LEDs (λmax = 462 nm; λ_1/2_ = 453 nm, and 473 nm) in the previous study [22], but we used turquoise green LEDs in the present study, because of their wider spectroscopic properties.

Two days before sampling, 2–5 fish were transferred to clear plastic cups (250 mL water in a 400 mL cup). No food was given from the day before sampling. Eyeballs were collected every 2 h starting from ZT1 to ZT23 under the entrained light–dark cycle (LD or SD) or from projected ZT1 (pZT1) to pZT23 on the first day in DD (LD-DD or SD-DD). The fish were anesthetized with ice. During the light and dark periods, sampling was conducted under a white fluorescent lamp (85 ± 8.5 μW cm^−2^, FHF32EX-N-HX-S, NEC) and a dim red light [22], respectively. The collected eyeballs were quickly homogenized with TRIzol reagent (Invitrogen).

### RNA extraction and cDNA synthesis

Total RNA was extracted from the eyeball using TRIzol Reagent (Invitrogen), and the quality was checked spectroscopically at 260 nm and 280 nm. Contaminating genomic DNA was removed using DNase treatment with RNase-free Recombinant DNase I (TaKaRa). cDNA was synthesized from 1 μg of total RNA using a High Capacity cDNA Reverse Transcription Kit (Applied Biosystems). Samples without reverse transcriptase were prepared as the negative control.

### Quantitative RT-PCR

The cDNA (20 μL) was diluted tenfold, of which 4 μL were used for qRT-PCR. The synthesized cDNA (4 μL), 5 μL of 2 × Fast SYBR Green Master Mix (Applied Biosystems), primers (500 nM, Tables 2 and 3), and distilled water were mixed up to 10 μL. qRT-PCR was performed using StepOnePlus (Applied Biosystems) with the following cycle conditions: 20 s at 95 °C, then 40 cycles of 3 s at 95 °C, and 30 s at 60 °C, and then 15 s at 95 °C, 60 s at 60 °C and 15 s at 95 °C (melting curve analysis). The relative expression levels of the *Cry* genes were determined using the ΔΔCT method [47]. The products were electrophoresed on a 3% agarose gel.

### Molecular phylogenetic analysis

A molecular phylogenetic tree of the CRY family was created using the neighbor-joining method (NJ method) using ClustalW ver. 2.1. Bootstrap probabilities were estimated from 1000 replicates.

### Statistical processing and harmonic analysis

RStudio (ver. 1.0.103) (https://rstudio.com/products/rstudio/) and SPSS (IBM ver. 28.0.0.0) were used for statistical analysis. Since we had small sample sizes at each sampling point (*n* = 3–5), the Kruskal–Wallis test and Dann-Bonferroni post-hoc test were performed for comparison between mRNA levels at different time points under each light condition. For the same reason, we did not compare mRNA levels at specific time points. Instead, we compared averaged *Cry* mRNA levels under different light conditions: A Shapiro–Wilk test was performed and showed that the distribution of the averaged *Cry* mRNA levels under most conditions departed significantly from normality. Based on this outcome, a non-parametric Mann–Whitney U test was performed. Results were considered statistically significant at *p* < 0.05. Error bars were used to indicate standard deviation. A cosinor fitting of the expression profile was performed using CircWave (ver. 1.4) by Hut, 2007, Groningen, The Netherlands. The peak times of gene expression were defined as acrophases. Numbers of sines in auto forward mode and *p*-values for wave fittings are summarized in Table S2.

## Supplementary Information

**Additional file 1: Supplementary Table S1.** Accession numbers of amino acid sequences used to construct the phylogenetic tree

**Additional file 2: Supplementary Table S2.** Cosinor fitting for data shown in Figures 2–4

**Additional file 3: Supplementary Tables S3–S5.** *p*-values in Kruskal-Wallis test for data shown in Figures 2–4

**Additional file 4: Supplementary Tables S6–S8.** *p*-values in Mann-Whitney U test for data shown in Figures 2–4

**Additional file 5: Supplementary Figure S1.** Emission spectra of light-emitting diode (LED)

**Additional file 6: Supplementary Figures S2–S13.** Cry expression profiles shown with statistical results

## Data Availability

All data generated or analyzed during this study are included in this published article and its supplementary information files.

## References

[1] Hamner WM (1963). Diurnal rhythm and photoperiodism in testicular recrudescence of the house finch. Science.

[2] Gwinner E (1996). Circadian and circannual programmes in avian migration. J Exp Biol.

[3] Dawson A, King VM, Bentley GE, Ball GF (2001). Photoperiodic control of seasonality in birds. J Biol Rhythms.

[4] Darrow JM, Duncan MJ, Bartke A, Bona-Gallo A, Goldman BD (1988). Influence of photoperiod and gonadal steroids on hibernation in the European hamster. J Comp Physiol A.

[5] Bunning E (1960). Circadian rhythms and the time measurement in photoperiodism. Cold Spring Harb Symp Quant Biol.

[6] Pittendrigh CS, Minis DH. The entrainment of circadian oscillations by light and their role as photoperiodic clocks. Am Nat. 1964;98:261–94. 10.1086/282327.

[7] Dunlap JC (1999). Molecular bases for circadian clocks. Cell.

[8] Shearman LP, Sriram S, Weaver DR, Maywood ES, Chaves I, Zheng B (2000). Interacting molecular loops in the mammalian circadian clock. Science.

[9] Ueda HR, Hayashi S, Chen W, Sano M, Machida M, Shigeyoshi Y (2005). System-level identification of transcriptional circuits underlying mammalian circadian clocks. Nat Genet.

[10] Tu DC, Batten ML, Palczewski K, Van Gelder RN (2004). Nonvisual Photoreception in the Chick Iris. Science.

[11] Ahmad M, Cashmore AR. HY4 gene of A. thaliana encodes a protein with characteristics of a blue-light photoreceptor. Nature. 1993;366(6451):162-6. 10.1038/366162a0. 10.1038/366162a0 8232555

[12] Stanewsky R, Kaneko M, Emery P, Beretta B, Wager-Smith K, Kay SA (1998). The cryb mutation identifies cryptochrome as a circadian photoreceptor in Drosophila. Cell.

[13] Ozturk N, Selby CP, Song S-H, Ye R, Tan C, Kao Y-T (2009). Comparative photochemistry of animal type 1 and type 4 cryptochromes. Biochemistry.

[14] Watari R, Yamaguchi C, Zemba W, Kubo Y, Okano K, Okano T (2012). Light-dependent structural change of chicken retinal cryptochrome4. J Biol Chem.

[15] Mitsui H, Maeda T, Yamaguchi C, Tsuji Y, Watari R, Kubo Y (2015). Overexpression in yeast, photocycle, and in vitro structural change of an avian putative magnetoreceptor cryptochrome4. Biochemistry.

[16] Otsuka H, Mitsui H, Miura K, Okano K, Imamoto Y, Okano T (2020). Rapid Oxidation Following Photoreduction in the Avian Cryptochrome4 Photocycle. Biochemistry.

[17] Liu C, Hu J, Qu C, Wang L, Huang G, Niu P (2015). Molecular evolution and functional divergence of zebrafish (Danio rerio) cryptochrome genes. Sci Rep.

[18] Kobayashi Y, Ishikawa T, Hirayama J, Daiyasu H, Kanai S, Toh H (2000). Molecular analysis of zebrafish photolyase/cryptochrome family: two types of cryptochromes present in zebrafish. Genes Cells.

[19] Meyer A, Van De Peer Y (2005). From 2R to 3R: Evidence for a fish-specific genome duplication (FSGD). BioEssays.

[20] Oliveri P, Fortunato AE, Petrone L, Ishikawa-Fujiwara T, Kobayashi Y, Todo T (2014). The cryptochrome/photolyase family in aquatic organisms. Mar Genomics.

[21] Ishikawa T, Hirayama J, Kobayashi Y, Todo T (2002). Zebrafish CRY represses transcription mediated by CLOCK-BMAL heterodimer without inhibiting its binding to DNA. Genes Cells.

[22] Okano K, Saratani Y, Tamasawa A, Shoji Y, Toda R, Okano T (2020). A photoperiodic time measurement served by the biphasic expression of Cryptochrome1ab in the zebrafish eye. Sci Rep.

[23] Matos-Cruz V, Blasic J, Nickle B, Robinson PR, Hattar S, Halpern ME (2011). Unexpected diversity and photoperiod dependence of the zebrafish melanopsin system. PLoS ONE.

[24] Brown C, Wolfenden D, Sneddon L. Goldfish (Carassius auratus). in Companion Animal Care and Welfare. 2018;467–78. 10.1002/9781119333708.ch23.

[25] Chen D, Zhang Q, Tang W, Huang Z, Wang G, Wang Y (2020). The evolutionary origin and domestication history of goldfish (Carassius auratus). Proc Natl Acad Sci.

[26] Hirai N, Torii Y, Matsuoka H, Ishii M. Genetic diversity and intrusion of alien populations of Oryzias latipes in Osaka Prefecture, central Japan. Japanese J Environ Entomol Zool. 2017;28(2):47–54. 10.11257/jjeez.28.47.

[27] Razani H, Hanyu I, Aida K (1987). Critical daylength and temperature level for photoperiodism in gonadal maturation of goldfish. Exp Biol.

[28] Urasaki H (1976). The role of pineal and eyes in the photoperiodic effect on the gonad of the medaka. Oryzias latipes Chronobiologia.

[29] Engeszer RE, Pattersion AB, Rao AA, Parichy DM (2007). Zebrafish in the wild: A review of natural history and new notes from the field. Zebrafish.

[30] Wang Y, Chen J, Zhu F, Hong Y (2017). Identification of medaka magnetoreceptor and cryptochromes. Sci China Life Sci.

[31] Shaw J, Boyd A, House M, Woodward R, Mathes F, Cowin G, el al. Magnetic particle-mediated magnetoreception. J R Soc Interface. 2015;12(110):0499. 10.1098/rsif.2015.0499. 10.1098/rsif.2015.0499 PMC4614459 26333810

[32] Cermakian N, Pando MP, Thompson CL, Pinchak AB, Selby CP, Gutierrez L (2002). Light induction of a vertebrate clock gene involves signaling through blue-light receptors and MAP kinases. Curr Biol.

[33] Tamai TK, Young LC, Whitmore D (2007). Light signaling to the zebrafish circadian clock by cryptochrome 1a. Proc Natl Acad Sci USA.

[34] Pittendrigh CS (1972). Circadian surfaces and the diversity of possible roles of circadian organization in photoperiodic induction. Proc Natl Acad Sci.

[35] Ono H, Hoshino Y, Yasuo S, Watanabe M, Nakane Y, Murai A (2008). Involvement of thyrotropin in photoperiodic signal transduction in mice. Proc Natl Acad Sci USA.

[36] Nishiwaki-Ohkawa T, Yoshimura T (2016). Molecular basis for regulating seasonal reproduction in vertebrates. J Endocrinol.

[37] Yoshimura T, Yasuo S, Watanabe M, Iigo M, Yamamura T, Hirunagi K (2003). Light-induced hormone conversion of T4 to T3 regulates photoperiodic response of gonads in birds. Nature.

[38] Nakane Y, Ikegami K, Ono H, Yamamoto N, Yoshida S, Hirunagi K (2010). A mammalian neural tissue opsin (Opsin 5) is a deep brain photoreceptor in birds. Proc Natl Acad Sci USA.

[39] Nakane Y, Ikegami K, Iigo M, Ono H, Takeda K, Takahashi D (2013). The saccus vasculosus of fish is a sensor of seasonal changes in day length. Nat Commun.

[40] Bradford CS, Miller AE, Toumadje A, Nishiyama K, Shirahata S, Barnes DW (1997). Characterization of cell cultures derived from Fugu. Japanese pufferfish Mol Mar Biol Biotechnol.

[41] Okano K, Ozawa S, Sato H, Kodachi S, Ito M, Miyadai T (2017). Light- and circadian-controlled genes respond to a broad light spectrum in Puffer Fish-derived Fugu eye cells. Sci Rep.

[42] Alerstam T, Gudmundsson GA, Green M, Hedenström A (2001). Migration along orthodromic sun compass routes by arctic birds. Science.

[43] Beer K, Helfrich-Förster C (2020). Model and non-model insects in chronobiology. Front Behav Neurosci.

[44] Mouritsen H, Atema J, Kingsford MJ, Gerlach G (2013). Sun compass orientation helps coral reef fish larvae return to their natal reef. PLoS ONE.

[45] Faillettaz R, Blandin A, Paris CB, Koubbi P, Irisson JO (2015). Sun-compass orientation in mediterranean fish larvae. PLoS ONE.

[46] Roberts NW, Needham MG (2007). A mechanism of polarized light sensitivity in cone photoreceptors of the goldfish Carassius auratus. Biophys J.

[47] Livak KJ, Schmittgen TD (2001). Analysis of relative gene expression data using real-time quantitative PCR and the 2(-Delta Delta C(T)) method. Methods.

