# Supplementary material for: Midnight/midday-synchronized expression of cryptochrome genes in the eyes of three teleost species, zebrafish, goldfish, and medaka
Source: Zoological Lett. 2022 Jun 7;8:8. doi: 10.1186/s40851-022-00192-4 (PMC9172026; doi:10.1186/s40851-022-00192-4)
Supplement: Supplementary file 5 — Additional file 5: Supplementary Figure S1. Emission spectra of light-emitting diode (LED) [file 40851_2022_192_MOESM5_ESM.pdf]

Supplementary Figure S1

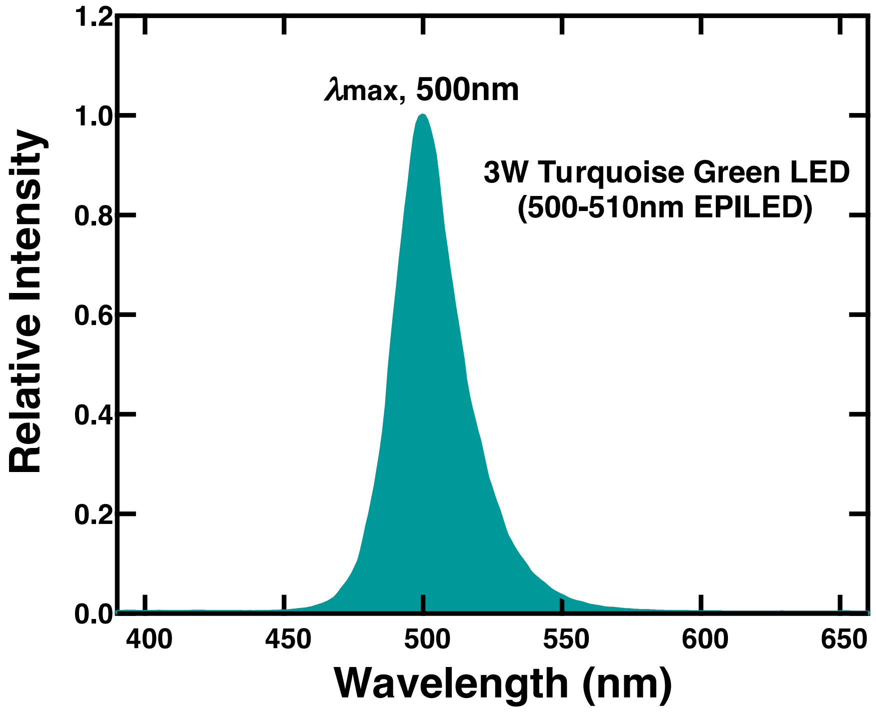

Fig. S1. Emission spectra of light-emitting diode (LED)
Turquoise LEDs (3W EPILED, 500-510nm, Futureeden, UK) were used for turquoise green light. The spectrum was measured using a hand-held spectrometer (UPRtek, MK-350N) and smoothened by Igor Pro (ver.8.04).
