# Supplementary material for: Midnight/midday-synchronized expression of cryptochrome genes in the eyes of three teleost species, zebrafish, goldfish, and medaka
Source: Zoological Lett. 2022 Jun 7;8:8. doi: 10.1186/s40851-022-00192-4 (PMC9172026; doi:10.1186/s40851-022-00192-4)
Supplement: Supplementary file 6 — Additional file 6: Supplementary Figures S2–S13. Cry expression profiles shown with statistical results [file 40851_2022_192_MOESM6_ESM.pdf]

Supplementary Figure S2

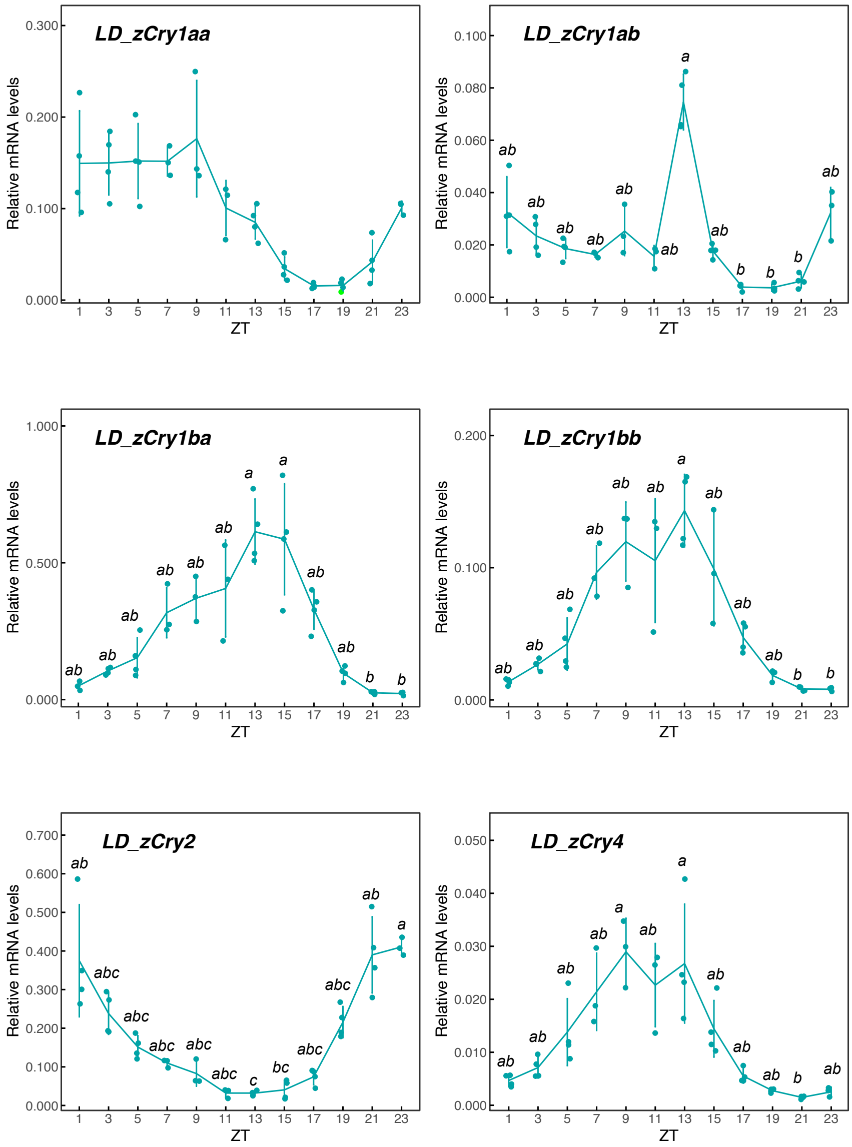

Fig. S2. *Cry* expression profiles in zebrafish eyes under long-day (LD) condition
Expression profiles of *zCry*s under LD condition (Figure 2) were shown with statistical results. Differences between groups were assessed by the Kruskal-Wallis test, followed by the Dann-Bonferroni post-hoc test for comparing multiple treatments. Different letters indicate statistically significant differences ($p < 0.05$) between groups. Expression levels of each mRNA were calculated relative to the synergistic mean of *zEf1α* and *zGapdh* expression levels. Error bars indicate standard deviation.

Supplementary Figure S3

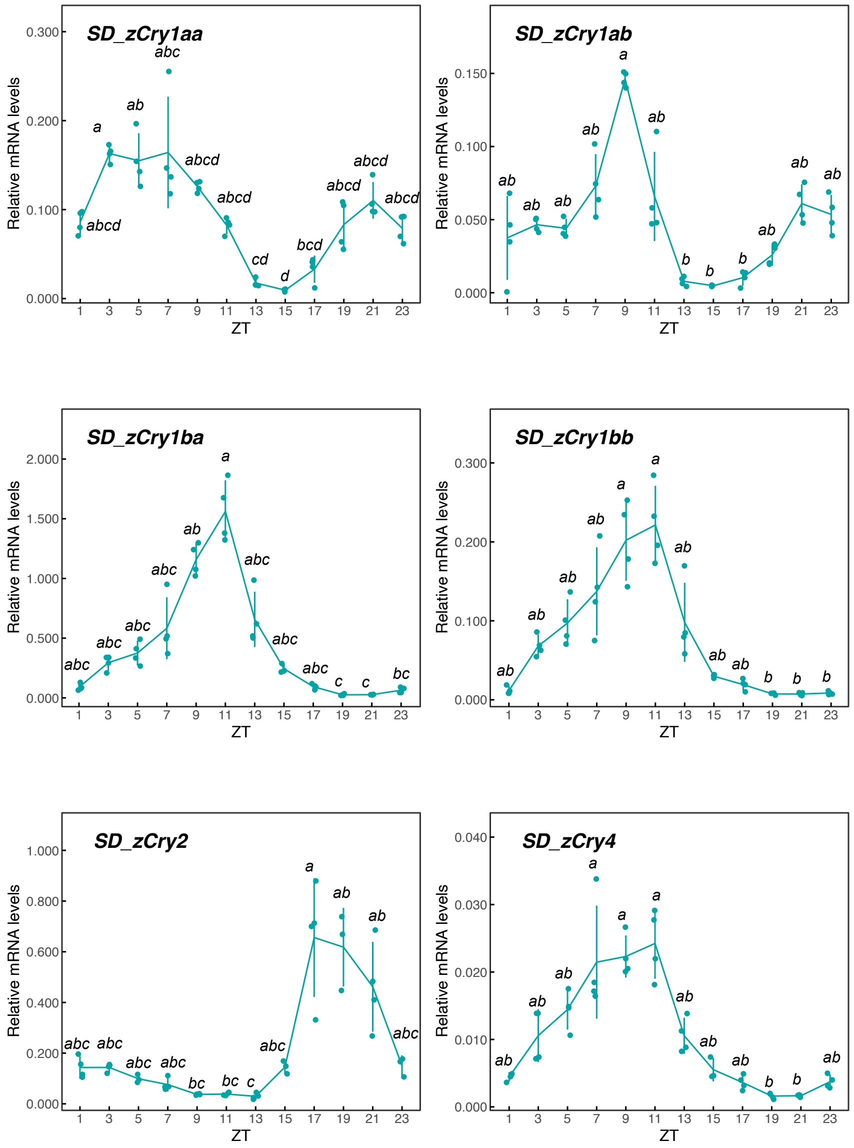

Figure S3 *Cry* expression profiles in zebrafish eyes under short-day (SD) condition
Expression profiles of *zCry*s under SD condition (Figure 2) were shown with statistical results. Differences between groups were assessed by the Kruskal-Wallis test, followed by the Dann-Bonferroni post-hoc test for comparing multiple treatments. Different letters indicate statistically significant differences ($p < 0.05$) between groups. Expression levels of each mRNA were calculated relative to the synergistic mean of *zEf1α* and *zGapdh* expression levels. Error bars indicate standard deviation.

Supplementary Figure S4

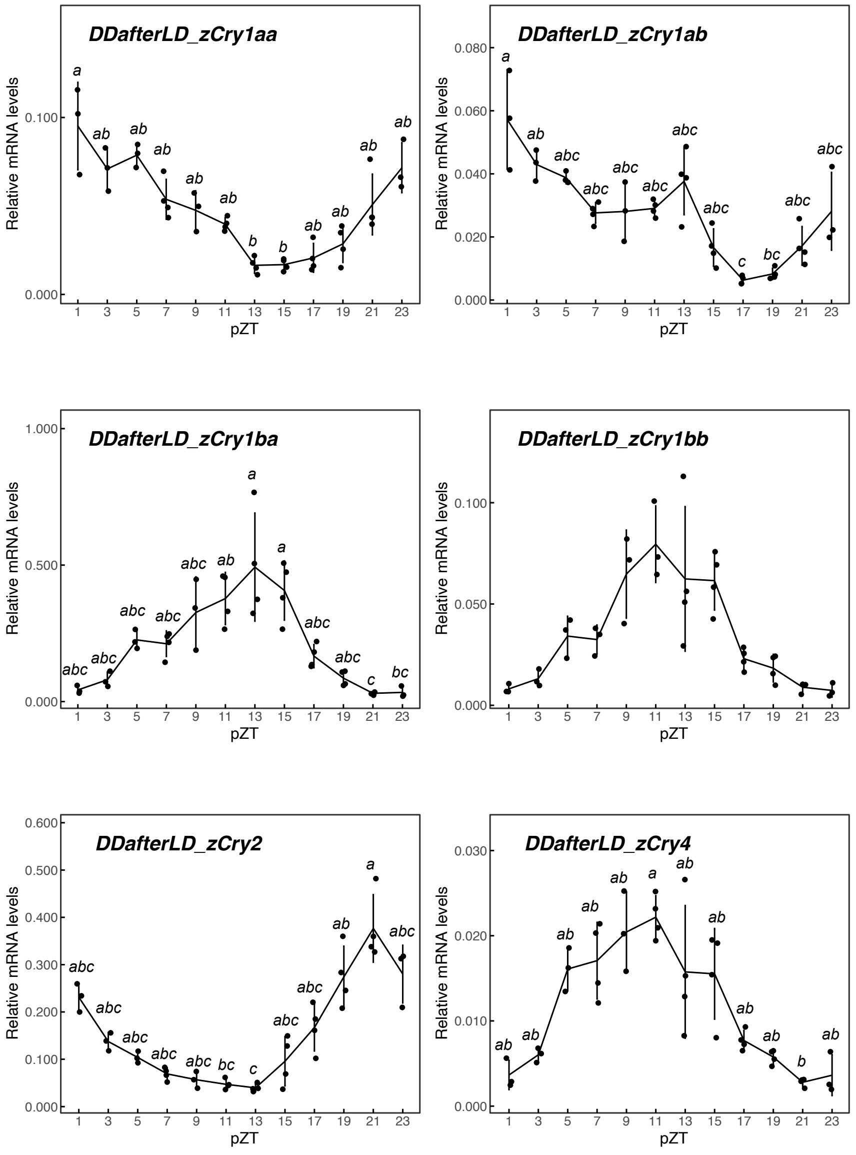

Figure S4 *Cry* expression profiles in zebrafish eyes under constant dark after long-day (LD-DD) condition

Expression profiles of *zCry*s in DD on the first day after LD entrainment (LD-DD; Figure 2) were shown with statistical results. Differences between groups were assessed by the Kruskal-Wallis test, followed by the Dann-Bonferroni post-hoc test for comparing multiple treatments. Different letters indicate statistically significant differences (p < 0.05) between groups. Expression levels of each mRNA were calculated relative to the synergistic mean of *zEf1α* and *zGapdh* expression levels. Error bars indicate standard deviation.

Supplementary Figure S5

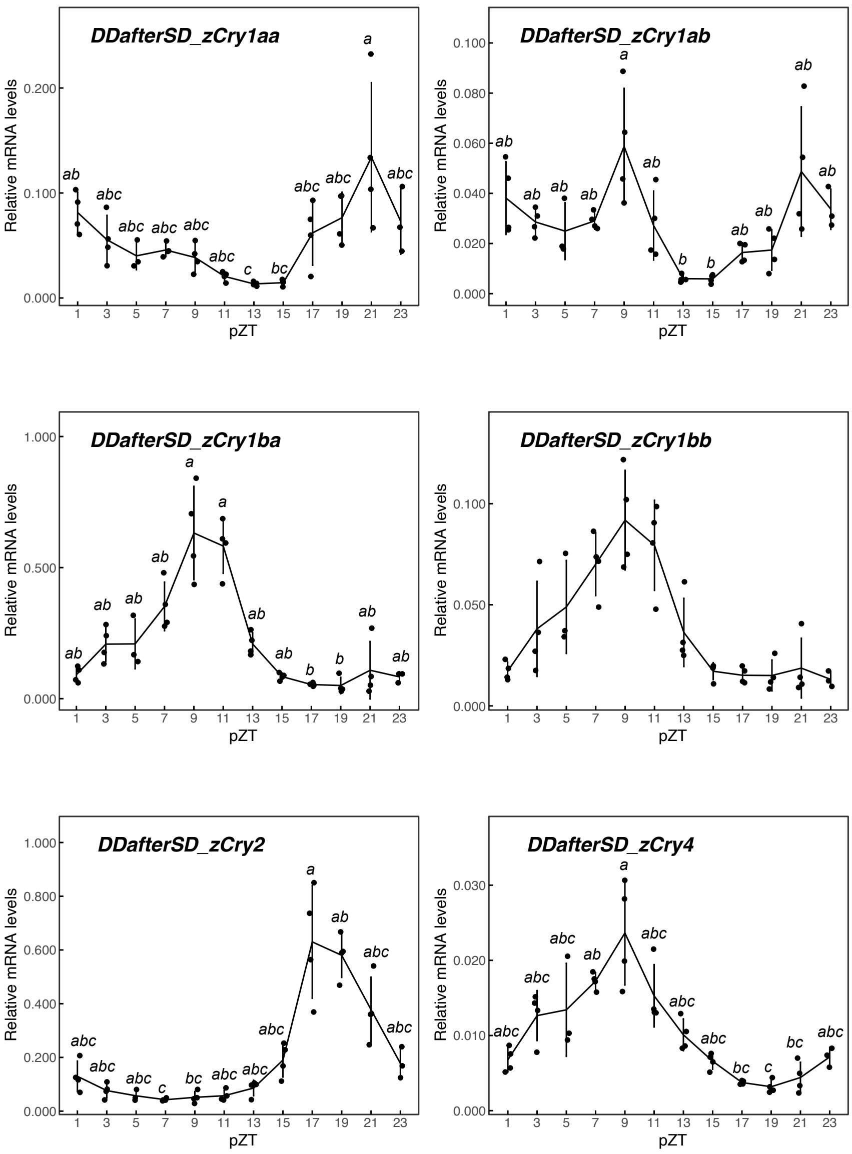

Figure S5 *Cry* expression profiles in zebrafish eyes under constant dark after short-day (SD-DD) condition

Expression profiles of *zCry*s in DD on the first day after SD entrainment (SD-DD; Figure 2) were shown with statistical results. Differences between groups were assessed by the Kruskal-Wallis test, followed by the Dann-Bonferroni post-hoc test for comparing multiple treatments. Different letters indicate statistically significant differences (p < 0.05) between groups. Expression levels of each mRNA were calculated relative to the synergistic mean of *zEf1α* and *zGapdh* expression levels. Error bars indicate standard deviation.

Supplementary Figure S6

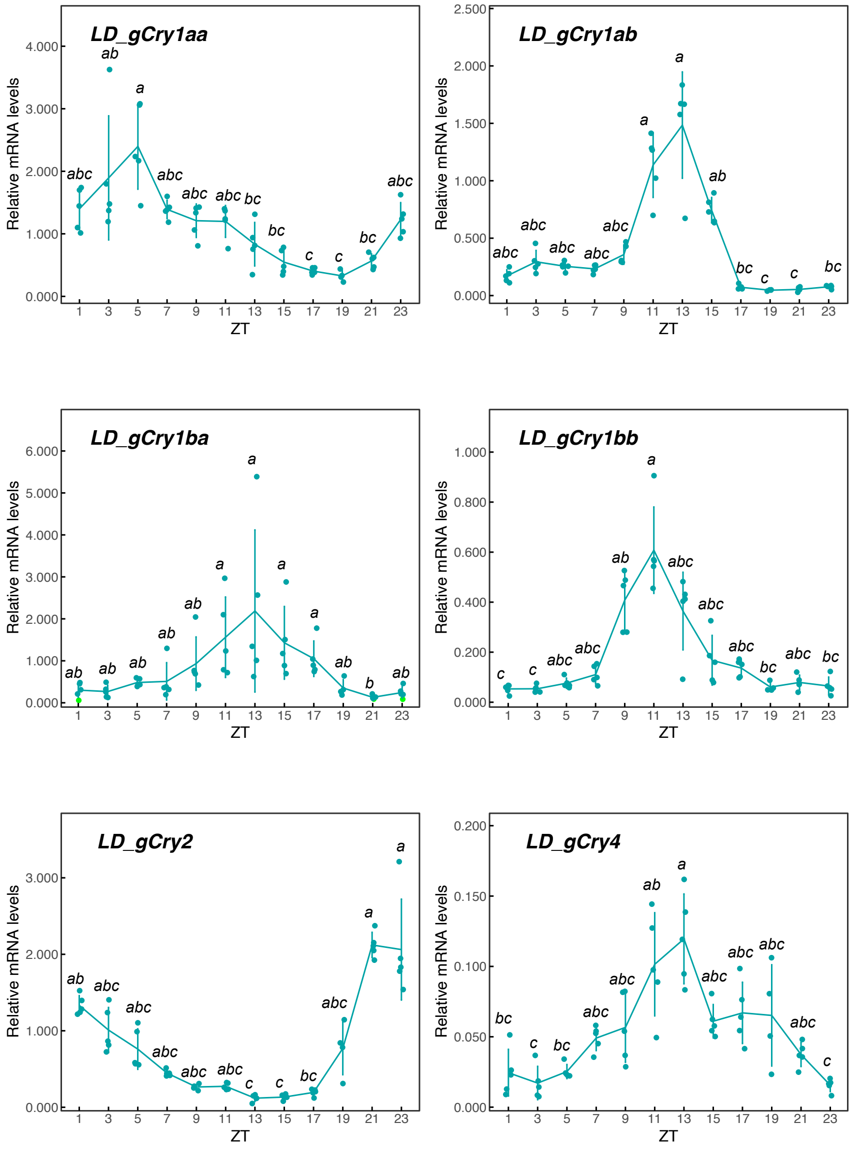

Figure S6 *Cry* expression profiles in goldfish eyes under long-day (LD) condition
Expression profiles of *gCry*s under LD condition (Figure 3) were shown with statistical results. Differences between groups were assessed by the Kruskal-Wallis test, followed by the Dann-Bonferroni post-hoc test for comparing multiple treatments. Different letters indicate statistically significant differences ($p < 0.05$) between groups. Expression levels of each mRNA were calculated relative to the synergistic mean of *gGusb, gPgk1,* and *gHprt1* expression levels. Error bars indicate standard deviation.

Supplementary Figure S7

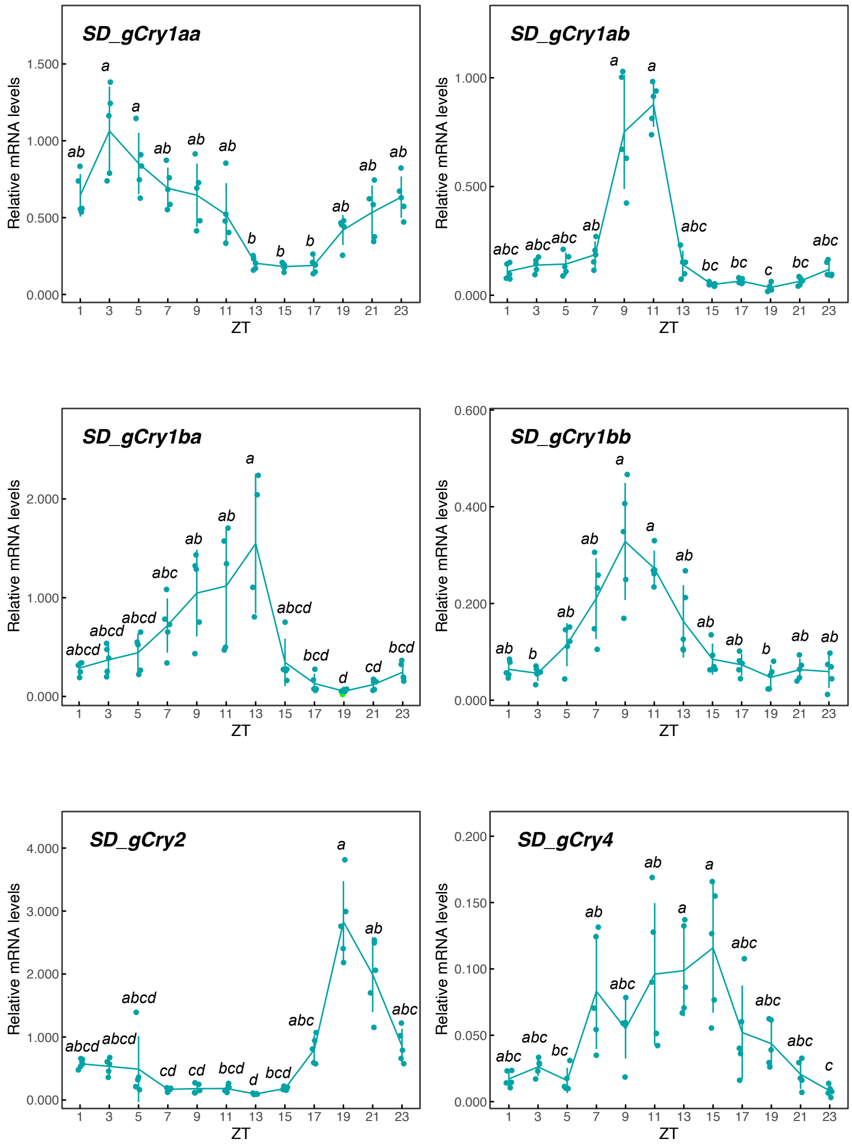

Figure S7 *Cry* expression profiles in goldfish eyes under short-day (SD) condition
Expression profiles of *gCry*s under SD condition (Figure 3) were shown with statistical results. Differences between groups were assessed by the Kruskal-Wallis test, followed by the Dann-Bonferroni post-hoc test for comparing multiple treatments. Different letters indicate statistically significant differences ($p < 0.05$) between groups. Expression levels of each mRNA were calculated relative to the synergistic mean of *gGusb, gPgk1,* and *gHprt1* expression levels. Error bars indicate standard deviation.

Supplementary Figure S8

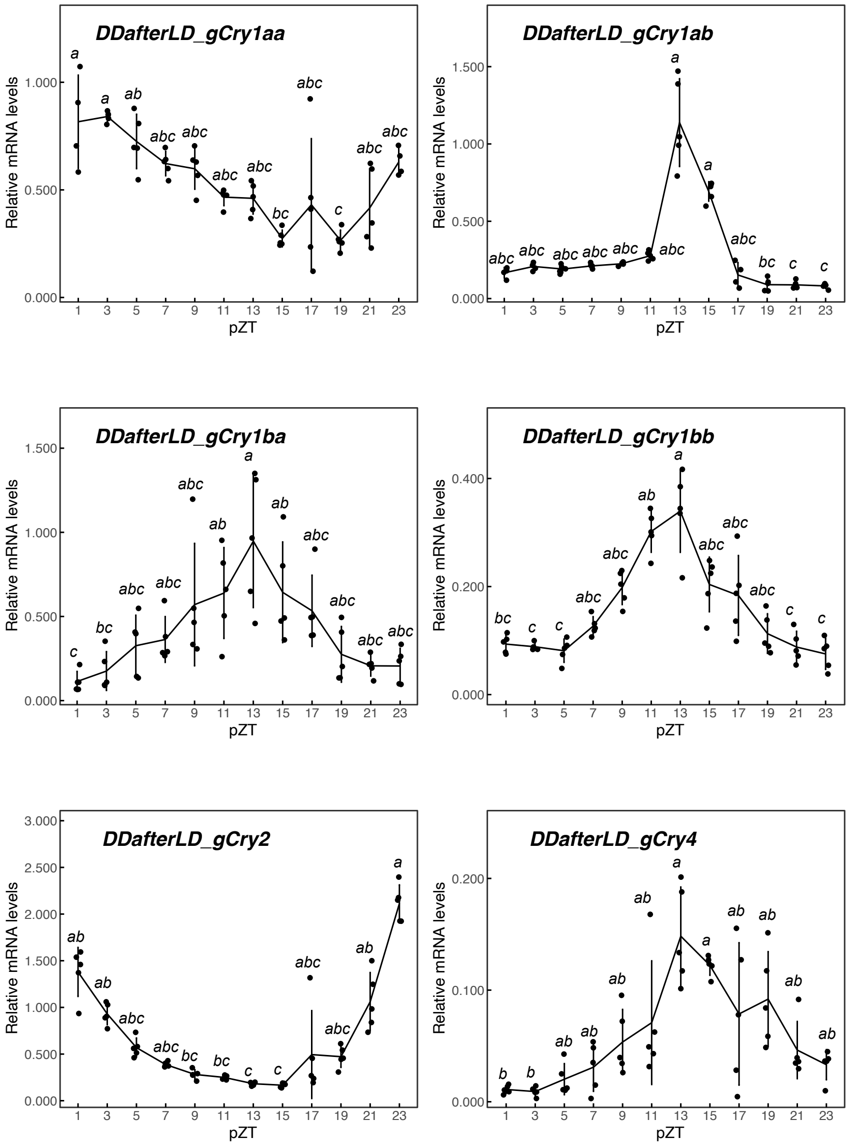

Figure S8 *Cry* expression profiles in goldfish eyes under constant dark after long-day (LD-DD) condition

Expression profiles of *gCry*s in DD on the first day after LD entrainment (LD-DD; Figure 3) were shown with statistical results. Differences between groups were assessed by the Kruskal-Wallis test, followed by the Dann-Bonferroni post-hoc test for comparing multiple treatments. Different letters indicate statistically significant differences (p < 0.05) between groups. Expression levels of each mRNA were calculated relative to the synergistic mean of *gGusb, gPgk1,* and *gHprt1* expression levels. Error bars indicate standard deviation.

Supplementary Figure S9

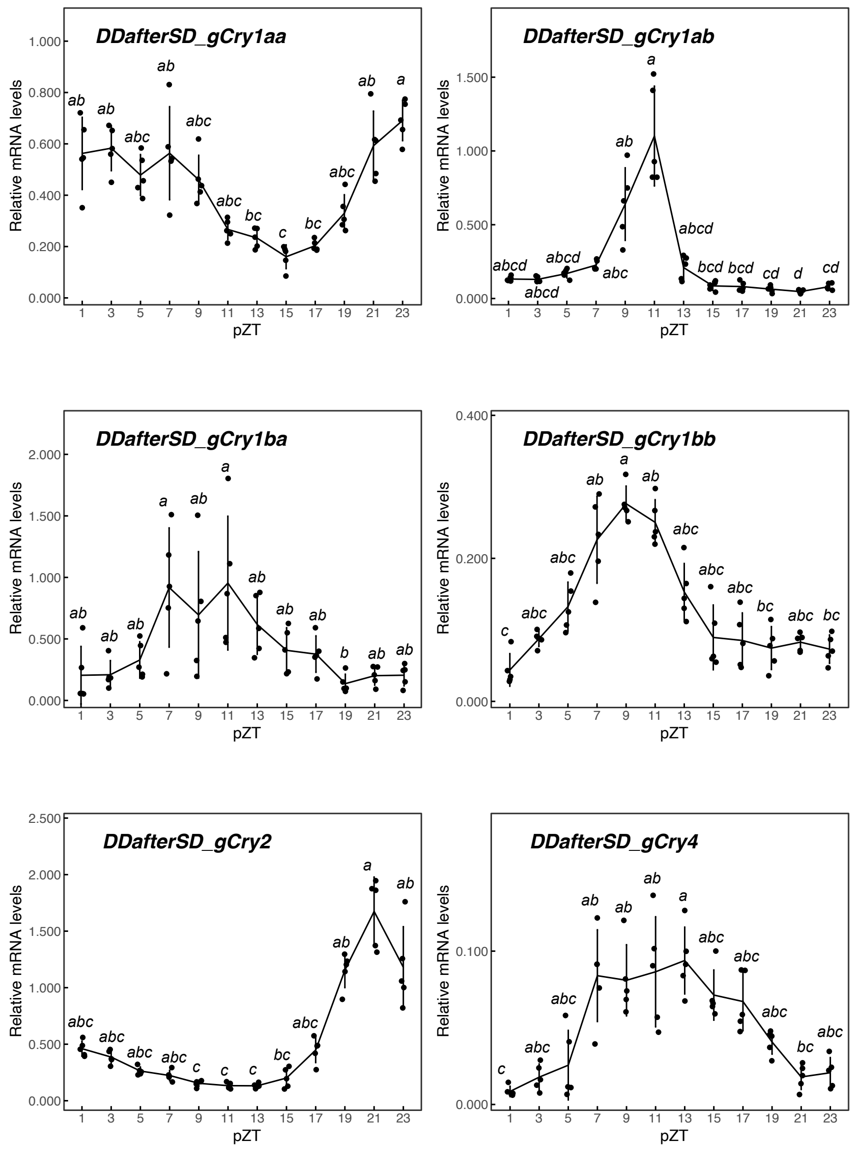

Figure S9 *Cry* expression profiles in goldfish eyes under constant dark after short-day (SD-DD) condition

Expression profiles of *gCry*s in DD on the first day after SD entrainment (SD-DD; Figure 3) were shown with statistical results. Differences between groups were assessed by the Kruskal-Wallis test, followed by the Dann-Bonferroni post-hoc test for comparing multiple treatments. Different letters indicate statistically significant differences ($p < 0.05$) between groups. Expression levels of each mRNA were calculated relative to the synergistic mean of *gGusb, gPgk1,* and *gHprt1* expression levels. Error bars indicate standard deviation.

Supplementary Figure S10

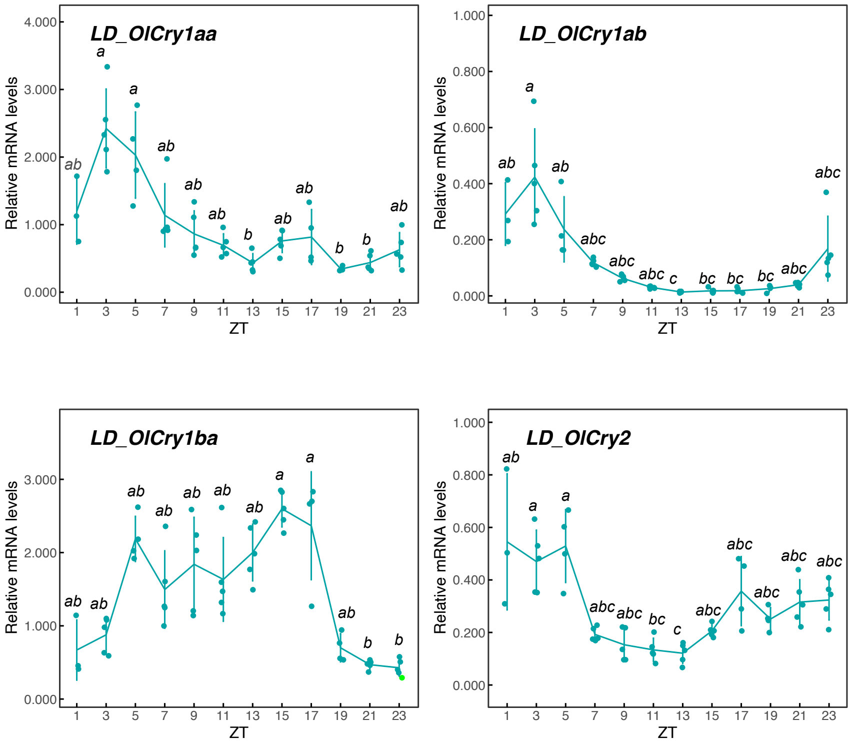

Figure S10 *Cry* expression profiles in medaka eyes under long-day (LD) condition
Expression profiles of *OlCry*s under LD condition (Figure 4) were shown with statistical results. Differences between groups were assessed by the Kruskal-Wallis test, followed by the Dann-Bonferroni post-hoc test for comparing multiple treatments. Different letters indicate statistically significant differences (p < 0.05) between groups. Expression levels of each mRNA were calculated relative to the synergistic mean of *OlGusb, OlEf1α* and *OlHprt1* expression levels. Error bars indicate standard deviation.

Supplementary Figure S11

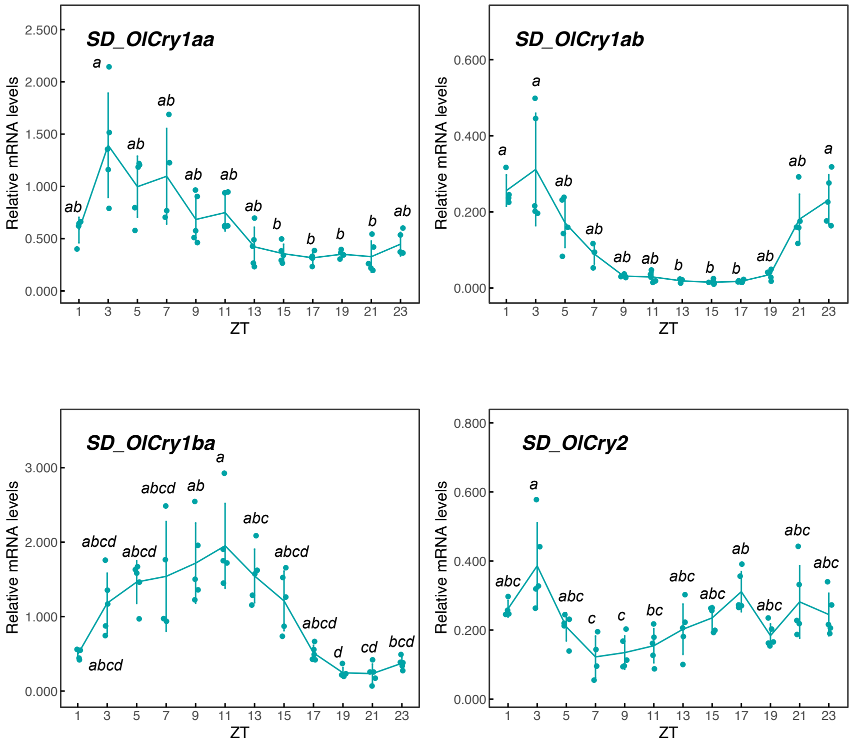

Figure S11 *Cry* expression profiles in medaka eyes under short-day (SD) condition
Expression profiles of *OlCry*s under SD condition (Figure 4) were shown with statistical results. Differences between groups were assessed by the Kruskal-Wallis test, followed by the Dann-Bonferroni post-hoc test for comparing multiple treatments. Different letters indicate statistically significant differences (p < 0.05) between groups. Expression levels of each mRNA were calculated relative to the synergistic mean of *OlGusb, OlEf1α* and *OlHprt1* expression levels. Error bars indicate standard deviation.

Supplementary Figure S12

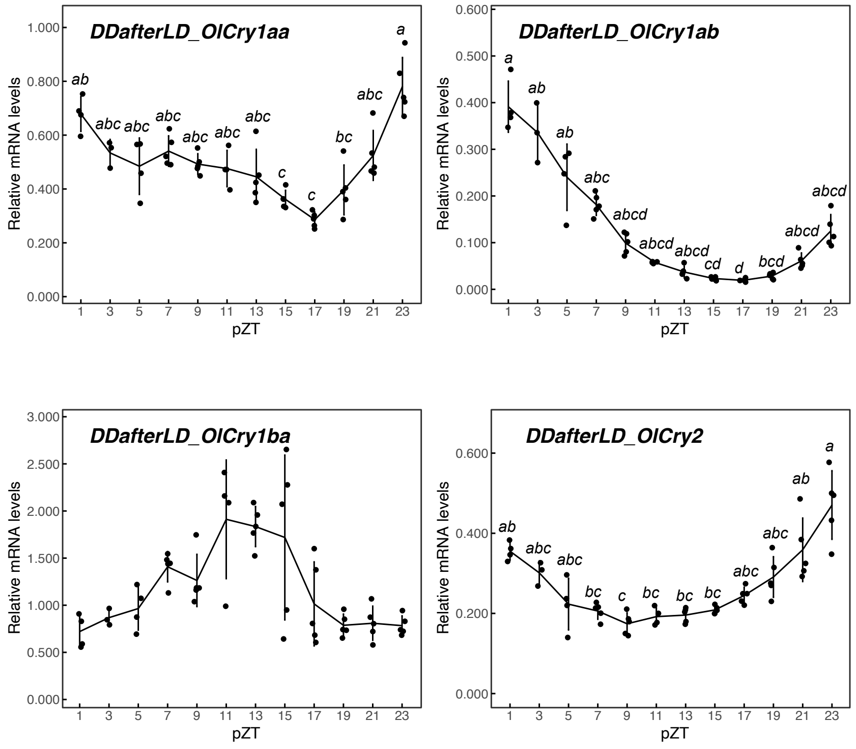

Figure S12 *Cry* expression profiles in medaka eyes under constant dark after long-day (LD-DD) condition

Expression profiles of *OlCry*s in DD on the first day after LD entrainment (LD-DD; Figure 4) were shown with statistical results. Differences between groups were assessed by the Kruskal-Wallis test, followed by the Dann-Bonferroni post-hoc test for comparing multiple treatments. Different letters indicate statistically significant differences (p < 0.05) between groups. Expression levels of each mRNA were calculated relative to the synergistic mean of *OlGusb, OlEf1α* and *OlHprt1* expression levels. Error bars indicate standard deviation.

Supplementary Figure S13

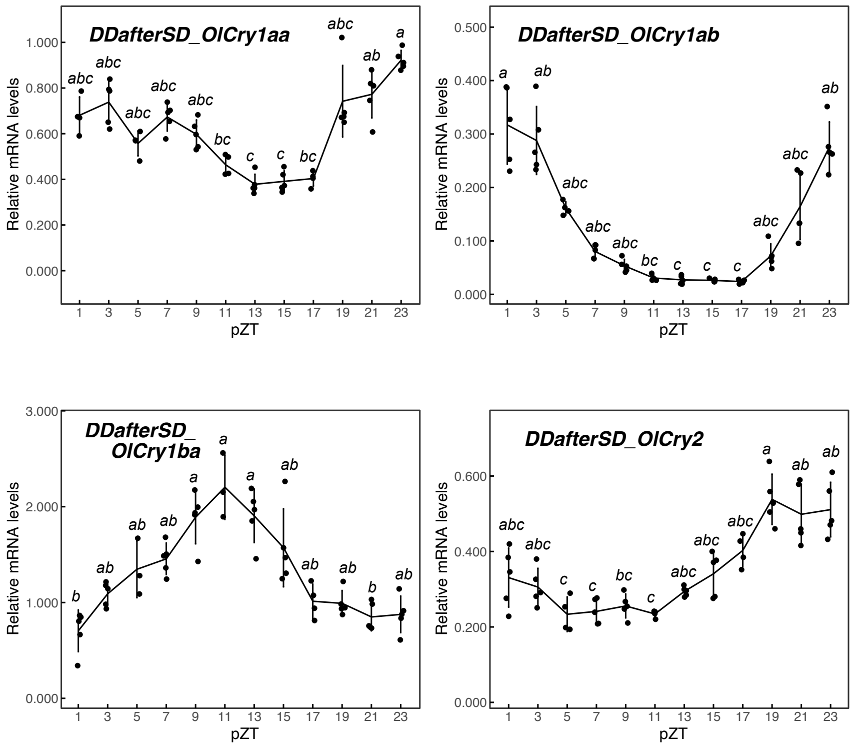

Figure S13 *Cry* expression profiles in medaka eyes under constant dark after short-day (SD-DD) condition

Expression profiles of *OlCry*s in DD on the first day after SD entrainment (SD-DD; Figure 4) were shown with statistical results. Differences between groups were assessed by the Kruskal-Wallis test, followed by the Dann-Bonferroni post-hoc test for comparing multiple treatments. Different letters indicate statistically significant differences ($p < 0.05$) between groups. Expression levels of each mRNA were calculated relative to the synergistic mean of *OlGusb, OlEf1α* and *OlHprt1* expression levels. Error bars indicate standard deviation.
